# Artery Tertiary Lymphoid Organs Control Aorta Immunity and Protect against Atherosclerosis via Vascular Smooth Muscle Cell Lymphotoxin β Receptors

**DOI:** 10.1016/j.immuni.2015.05.015

**Published:** 2015-06-16

**Authors:** Desheng Hu, Sarajo K. Mohanta, Changjun Yin, Li Peng, Zhe Ma, Prasad Srikakulapu, Gianluca Grassia, Neil MacRitchie, Gary Dever, Peter Gordon, Francis L. Burton, Armando Ialenti, Suleman R. Sabir, Iain B. McInnes, James M. Brewer, Paul Garside, Christian Weber, Thomas Lehmann, Daniel Teupser, Livia Habenicht, Michael Beer, Rolf Grabner, Pasquale Maffia, Falk Weih, Andreas J.R. Habenicht

**Affiliations:** 1 Institute of Molecular Immunology, Helmholtz Zentrum München, Marchioninistrasse 25, 81377 Munich, Germany; 2 Leibniz Institute for Age Research, Fritz Lipmann-Institute, 07745 Jena, Germany; 3 Institute for Cardiovascular Prevention, Ludwig-Maximilians University, Pettenkoferstrasse 9, 80336 Munich, Germany; 4 Department of Traditional Chinese Medicine, Medical College of Xiamen University, Xiamen University, 361102 Xiamen, P.R. China; 5 Cardiovascular Research Center (CVRC), University of Virginia, 415 Lane Rd, Post Box 801394, Charlottesville, VA 22908, USA; 6 Centre for Immunobiology, Institute of Infection, Immunity & Inflammation, College of Medical, Veterinary and Life Sciences, University of Glasgow, Glasgow, G12 8TA, UK; 7 Department of Pharmacy, University of Naples Federico II, 80131 Naples, Italy; 8 Strathclyde Institute of Pharmacy & Biomedical Sciences, University of Strathclyde, Glasgow G4 0RE, UK; 9 Institute of Cardiovascular and Medical Sciences, University of Glasgow, G12 8TA, UK; 10 DZHK, German Center for Cardiovascular Research, Munich Heart Alliance, Pettenkoferstrasse 9, 80336 Munich, Germany; and Cardiovascular Research Institute Maastricht, Maastricht, the Netherlands; 11 Institute for Medical Statistics, University of Jena, Jena University Hospital, 07743 Jena, Germany; 12 Department for Laboratory Medicine, Ludwig-Maximilians-University, Marchioninistr. 15, 81377 Munich, Germany; 13 II. Medizinische Klinik und Poliklinik; Technische Universität Muenchen, Klinikum rechts der Isar, Ismaningerstrasse 22, 81675 Munich, Germany; 14 Department for Information Technology, University of Jena, Jena University Hospital, 07743 Jena, Germany

## Abstract

Tertiary lymphoid organs (TLOs) emerge during nonresolving peripheral inflammation, but their impact on disease progression remains unknown. We have found in aged *Apoe*^−/−^ mice that artery TLOs (ATLOs) controlled highly territorialized aorta T cell responses. ATLOs promoted T cell recruitment, primed CD4^+^ T cells, generated CD4^+^, CD8^+^, T regulatory (Treg) effector and central memory cells, converted naive CD4^+^ T cells into induced Treg cells, and presented antigen by an unusual set of dendritic cells and B cells. Meanwhile, vascular smooth muscle cell lymphotoxin β receptors (VSMC-LTβRs) protected against atherosclerosis by maintaining structure, cellularity, and size of ATLOs though VSMC-LTβRs did not affect secondary lymphoid organs: Atherosclerosis was markedly exacerbated in *Apoe*^*−/−*^*Ltbr*^*−/−*^ and to a similar extent in aged *Apoe*^−/−^*Ltbr*^fl/fl^*Tagln-cre* mice. These data support the conclusion that the immune system employs ATLOs to organize aorta T cell homeostasis during aging and that VSMC-LTβRs participate in atherosclerosis protection via ATLOs.

## Highlights

• Artery tertiary lymphoid organs control atherosclerosis T cell immunity
• Artery tertiary lymphoid organs generate effector memory T cells
• Artery tertiary lymphoid organs convert naive CD4^+^ T cells into induced Treg cells
• Artery tertiary lymphoid organs protect from atherosclerosis

## Graphical Abstract

**Figure:**
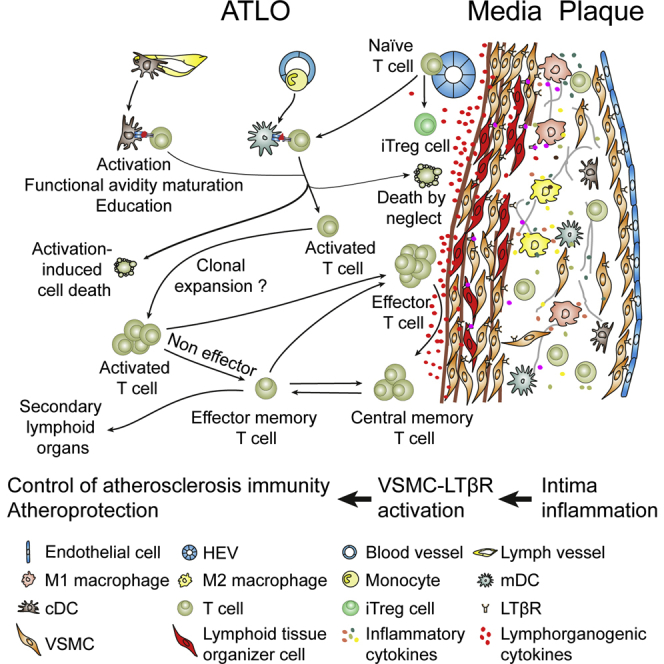

## Teaser

Tertiary lymphoid organs emerge during nonresolving inflammation. Habenicht and colleagues find that atherosclerosis immune responses are controlled by artery tertiary lymphoid organs in the adventitial connective tissue adjoining arteries. These lymphocyte aggregates arise through vascular smooth muscle cell lymphotoxin β receptor signaling and act as powerhouses of protective atherosclerosis immunity.

## Introduction

A central tenet in immunology is that primary T cell responses are initiated in secondary lymphoid organs (SLOs) (Mackay, 1999, Mackay and von Andrian, 2001, Sallusto et al., 2004, Steinman, 2012, Woodland and Kohlmeier, 2009). In contrast, roles of tertiary lymphoid organs (TLOs) have not yet been defined (Aloisi and Pujol-Borrell, 2006, Drayton et al., 2006, Moyron-Quiroz et al., 2004, Roozendaal and Mebius, 2011). Although similarities between SLOs and TLOs are apparent, major differences deserve attention: SLOs form during ontogeny at predetermined locations, trigger priming of naive T cells following interaction with dendritic cells (DCs), and resume quiescence upon elimination of antigen (Miller et al., 2004). In contrast, TLOs emerge as unencapsulated lymphoid aggregates in chronic inflammatory diseases at undetermined locations in adult organisms (Gräbner et al., 2009, Nathan and Ding, 2010, Weyand et al., 2001). Though TLO neogenesis correlates with disease severity (Galkina et al., 2006, Galkina and Ley, 2009, Gräbner et al., 2009, Lopez-Diego and Weiner, 2008, Moyron-Quiroz et al., 2004), their role has not been determined (Gräbner et al., 2009, Mohanta et al., 2014).

We have observed that artery TLOs (ATLOs) emerge in the aorta adventitia adjacent to atherosclerotic plaques in *Apoe*^*−/−*^ mice during aging and that their size and structure correlate with disease severity in a lymphotoxin β receptor (LTβR)-dependent way (Gräbner et al., 2009, Moos et al., 2005, Zhao et al., 2004). We have also noticed that vascular smooth muscle cells (VSMCs) of abdominal aorta segments that are located between atherosclerotic plaques and ATLOs express the lymphorganogenic cytokines, i.e., CCL21 and CXCL13 (Gräbner et al., 2009), that VSMCs express LTβRs in vivo, and that LTβR signaling initiates transdifferentiation of VSMCs to a lymphoid tissue organizer-like phenotype in vitro (Lötzer et al., 2010). These results are consistent with the view that media VSMC-LTβRs transduce plaque-derived inflammatory cues to the adventitia to promote ATLO neogenesis (Aloisi and Pujol-Borrell, 2006, Drayton et al., 2006, Gebhardt et al., 2011, Geginat et al., 2001, Glass and Witztum, 2001, Gräbner et al., 2009, Groom and Luster, 2011, Hammerschmidt et al., 2008, Hansson and Hermansson, 2011, Lichtman et al., 2013, Mohanta et al., 2014, Moyron-Quiroz et al., 2004, Nathan and Ding, 2010, Roozendaal and Mebius, 2011, Weber and Noels, 2011). In the present study, we explored the impact of ATLOs on atherosclerosis T cell responses and asked whether VSMC-LTβRs might participate in disease progression. Our data reveal that the aging immune system employs ATLOs to control atherosclerosis T cell immunity and that VSMC-LTβRs maintain ATLO structure and attenuate atherosclerosis.

## Results

### Systemic T Cell Aging in Wild-Type and *Apoe*^*−/−*^ Mice

T cell receptor-β^+^ (TCRβ^+^) cells per renal lymph node (RLN), spleen, and blood contracted by ∼50% during aging and the magnitude of contraction was similar in *Apoe*^*−/−*^ and WT mice (data not shown). Aging also altered the composition of T cell subtypes: CD4^+^ T cell frequencies decreased by ∼20%–30%, whereas CD4^+^Foxp3^+^ regulatory T (Treg) cells increased by ∼100% in SLOs and CD8^+^ T cells showed minor changes (Figures S1A and S2A). T cell activation and homing markers (Sheridan and Lefrançois, 2011) were analyzed on T cell subtypes: PD-1^+^ cells increased in all T cell subtypes, CD103^+^ cells increased in CD4^+^ and Treg cells but decreased in CD8^+^ cells, CD62L^+^ cells decreased in CD4^+^ and Treg cells, whereas they remained unchanged in CD8^+^ T cells; however, CD69^+^ and CXCR3^+^ cells increased in all T cell subtypes (Figures S1A and S2A). Again, aging-associated changes remained identical in *Apoe*^−/−^ versus WT mice. These data revealed large aging-associated changes in T cell subtype composition and activation, which was identical in *Apoe*^*−/−*^ and WT mice (see also Linton and Dorshkind, 2004, Montecino-Rodriguez et al., 2013).

MIAME-compliant microarrays of *Apoe*^−/−^ and WT spleens and blood (http://www.ncbi.nlm.nih.gov/geo/); deposited in the NCBI Gene Expression Omnibus (GEO); accession number GSE40156) revealed robust age-associated changes in total gene-expression profiles and in gene ontology (GO) terms related to immune and inflammatory responses but remained similar in *Apoe*^*−/−*^ versus WT mice (Figures S2D and S2E; Table S1) (C.Y. and A.J.R.H., unpublished data). Transcript profiles of WT aortas also showed age-associated changes (Figure S1B; Table S1). However, unlike SLOs and blood, aged *Apoe*^−/−^ versus WT aortas indicated extensive changes in a large number of overlapping and newly expressed transcripts (Figures S1B, S2B, and S2C; Table S1).

### mRNA Mapping of Tissue Microdomains Delineates the Territoriality of Atherosclerosis Immune Responses in the Aged Aorta

**Figure 1:**
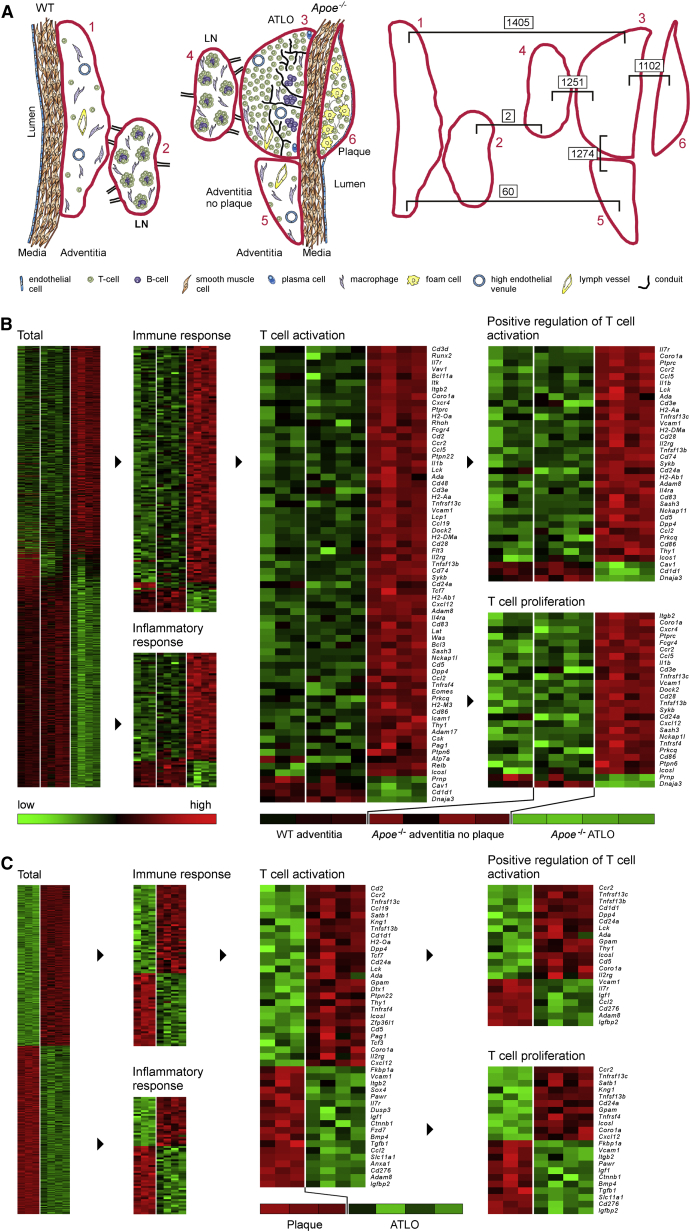
Transcript Atlases Reveal a High Degree of Territoriality of Gene Expression in the Arterial Wall (A) Anatomy of WT and *Apoe*^−/−^ aortas and RLNs (left) and the numbers of differentially expressed mRNAs in two-tissue comparisons (right) are shown of 78- to 85-week-old mice. Student’s t test with Benjamini-Hochberg correction; n = 3 WT and *Apoe*^−/−^ mice except n = 4 for *Apoe*^−/−^ adventitia no plaque and n = 4 for ATLOs. (B) Adventitia cluster show total differentially expressed genes (left) and mRNAs in respective GO terms (right). (C) Plaque-ATLO cluster is shown in respective GO terms (right). Cluster analyses were performed using ANOVA with Benjamini-Hochberg correction. Signal intensities and statistics are reported in Table S1 (see also Figure S3).

India ink in situ injections indicated that RLNs drain the abdominal aorta (data not shown). A transcriptome atlas of abdominal aortas and RLNs was constructed from aorta tissue microdomains using laser capture microdissection (LCM)-derived microarrays (Figure 1A) (C.Y. and A.J.R.H., unpublished data). Lesion development in *Apoe*^*−/−*^ mice is primarily a function of lipid accumulation, and inflammation is secondary. To assess the territoriality of inflammation and of immune responses in arterial wall laminae and their corresponding aorta-draining RLNs, we analyzed transcript atlases in detail. *Apoe*^−/−^ and WT RLN maps were virtually identical (Figure 1A, S3B, S3G, and S3H; Table S1) sharply contrasting with large numbers of differentially expressed transcripts in ATLOs versus WT adventitia, ATLOs versus *Apoe*^−/−^ adventitia without plaque, and ATLOs versus plaques (Figures 1A, S3A, S3C, and S3D; Table S1). More comprehensive information was obtained using three-tissue comparisons. The adventitia cluster showed large overlaps between WT adventitia and *Apoe*^−/−^ adventitia without plaque contrasting with transcripts expressed by adjacent ATLOs (Figures 1A, 1B, S3C, and S3D). The plaque-ATLO cluster indicated predominant expression of T cell-regulating genes in ATLOs versus inflammation-regulating genes in plaques (Figures 1C, S3E, and S3F) and the LN cluster revealed that ATLOs predominantly expressed inflammatory response-related genes when compared to RLNs (Figures S3B, S3G, and S3H).

### ATLOs Are T Memory Cell Homing Sites

**Figure 2:**
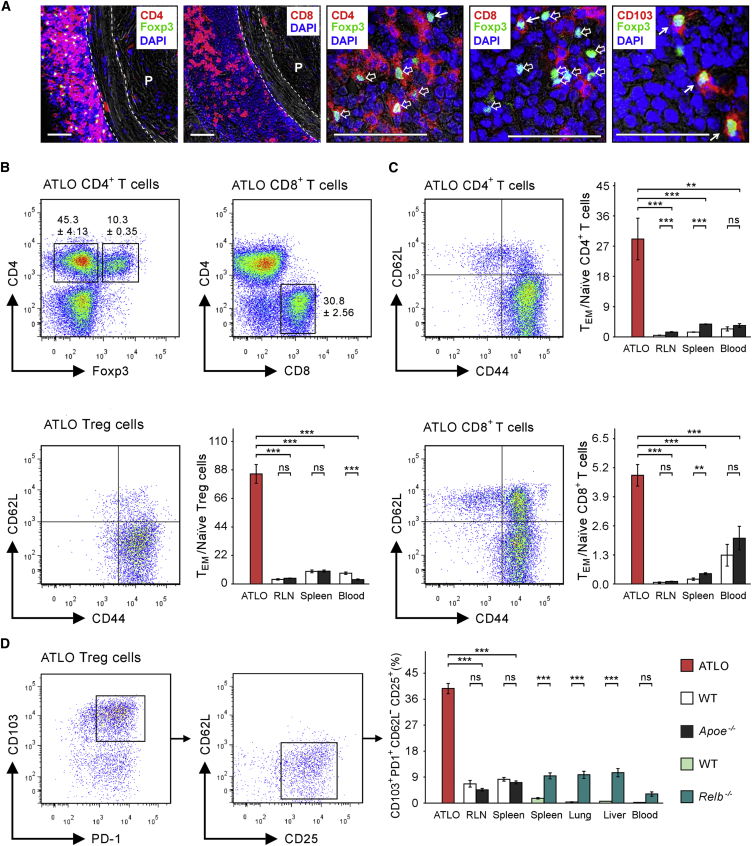
ATLOs Harbor Distinct Sets of TCRβ^+^ T Cell Subtypes (A) T cell abundance in ATLOs versus plaques. Immunofluorescence detection of 78- to 85-week-old *Apoe*^*−/−*^ CD4^+^ Treg cells, and CD8^+^ T cells in ATLOs versus plaques (P) (two left panels); CD4^+^ Treg cells (middle; open arrows); CD4^−^ Treg cells (middle; closed arrow); CD8^+^ Treg cells (second right; closed arrow); and CD103^+^ Treg cells (right; closed arrows) in T cell areas (n = 3 mice). Dotted lines indicate media. DAPI stains nuclei. Scale bars represent 50 μm for two left panels and 100 μm for three right panels. (B) Lymphocyte subsets in ATLOs. Flow cytometry plots show ATLO CD4^+^Foxp3^−^ T cells, CD4^+^Foxp3^+^ Treg cells (left), and CD8^+^ T cells (right) from the TCRβ^+^ cell gate of 78- to 85-week-old *Apoe*^*−/−*^ mice. (C) Naive and T_EM_ cells in ATLO T cell subsets. Abundance of T_EM_ cells (CD62L^−^CD44^+^), T_CM_ cells (CD62L^+^CD44^+^), naive cells (CD62L^+^CD44^−^) in CD4^+^ T cells, Treg cells, and CD8^+^ T cells in ATLOs versus RLNs, spleen, and blood of 78- to 85-week-old *Apoe*^*−/−*^ mice. (D) ATLO Treg cell phenotype. 40% of ATLO Treg cells are CD103^+^PD1^+^CD62L^−^CD25^+^ contrasting to those in 78- to 85-week-old WT and *Apoe*^−/−^ spleen and LN, and in 9- to 12-week-young WT and *Relb*^−/−^ spleen, lung, liver, and blood. Flow cytometry data are representative of three independent experiments with pooled one to two mice per genotype per experiment with two technical replicates (B and C), or with one mouse per genotype (D). Means ± SEM, and p values corrected for multiple testing (Bonferroni) were estimated using the GEE model. ^∗^p ≤ 0.05; ^∗∗^p ≤ 0.01; ^∗∗∗^p ≤ 0.001 (see also Figure S4).

The majority of aorta T cells in aged *Apoe*^−/−^ mice are present in the adventitia (Gräbner et al., 2009, Moos et al., 2005). ATLO T cells corresponded to CD4^+^, CD8^+^, CD4^+^ Treg cells, and few CD8^+^ Treg cells (Figures 2A and 2B). During primary immune responses in SLOs CD62L^−^CD44^+^ T effector memory (T_EM_) and CD62L^+^CD44^+^ T central memory (T_CM_) cells are generated from naive CD4^+^ T cells (Mackay, 1999, Mackay and von Andrian, 2001, Sallusto et al., 2004, Steinman, 2012, Woodland and Kohlmeier, 2009). Naive CD4^+^ T cells were rare in ATLOs (Figure 2C). Indeed, the large majority of ATLO CD4^+^ T cells were T_EM_ cells yielding a 27-fold ratio of T_EM_ versus naive cells (Figure 2C). Treg cells also had a T_EM_ phenotype yielding an effector memory Treg versus naive Treg cell ratio of 86 fold. Similar results were obtained for CD8^+^ T cells (Figure 2C). The composition of ATLO memory cells contrasted to that in SLOs and blood, which contained fewer T_EM_ cells (Figure 2C). There was no systemic alteration in any memory T cell subtype in liver, lung, and other peripheral tissues between the mouse genotypes (data not shown). These data showed that T_EM_ and T_CM_ T cells dominate in ATLOs when compared to their SLO counterparts.

### ATLOs Educate Aorta T Cells

Tissue-specific homing and education of T_EM_ and T_CM_ cells is essential for long-term immunosurveillance in peripheral tissues. To achieve effective immunosurveillance, the immune system educates memory cells to express functionally relevant tissue homing molecules (Lathrop et al., 2011, Lathrop et al., 2008, Mackay, 1999, Mackay and von Andrian, 2001, Mikhak et al., 2013, Sallusto et al., 2004). T cell tissue tropism has been studied in skin and intestine (Sheridan and Lefrançois, 2011), but little is known about atherosclerosis. We determined expression of prototypic homing and function-associated markers (Sheridan and Lefrançois, 2011). Sizable populations of ATLO CD4^+^ and CD8^+^ T cells were CD103^+^ and PD-1^+^ (Figures S4A and S4C). 70% of Treg cells expressed CD103 and 79% expressed PD-1 contrasting with ATLO CD8^+^ T cells (Figures S4B and S4C). While CD69 was expressed in 44% of ATLO CD4^+^ and CD8^+^ T cells, >80% of Treg cells expressed CD69 (data not shown). In contrast, CD103, CD69, and PD-1 were low on T cells in SLOs and blood and expression was identical or changes were small in *Apoe*^*−/−*^ versus WT mice (Figure S4; data not shown). When compared to SLOs, all ATLO T cell subtypes showed low expression of the chemokine receptors CXCR3, CCR4, and CCR7, as well as CD122, though comparably higher *Tnfrsf18* (tumor necrosis factor receptor superfamily member 18; also referred to as glucocorticoid-induced tumor necrosis factor receptor-related gene, i.e., GITR; data not shown). Thus, all T cell subtypes and in particular ATLO Treg cells showed strong atherosclerosis education signatures. The magnitude of T cell education in atherosclerosis is further illustrated by comparing ATLO Treg cell phenotypes with those in a mouse model of systemic multiorgan inflammation, i.e., *Relb*^*−/−*^ mice (Weih et al., 1995). Although *Relb*^*−/−*^ spleen, lung, liver, and blood showed increased numbers of CD103^+^PD1^+^CD62L^−^CD25^+^ Treg cells, they fell short of those in ATLOs by a large margin (Figure 2D). Moreover, non-obese diabetic (NOD) mouse SLOs and pancreases (i.e., a model of autoimmune diabetes) did not reveal educated T cells (data not shown).

### ATLOs Direct Naive CD4^+^ and Naive CD8^+^ T Cell Homing by Enhanced Recruitment and Decreased Egress

**Figure 3:**
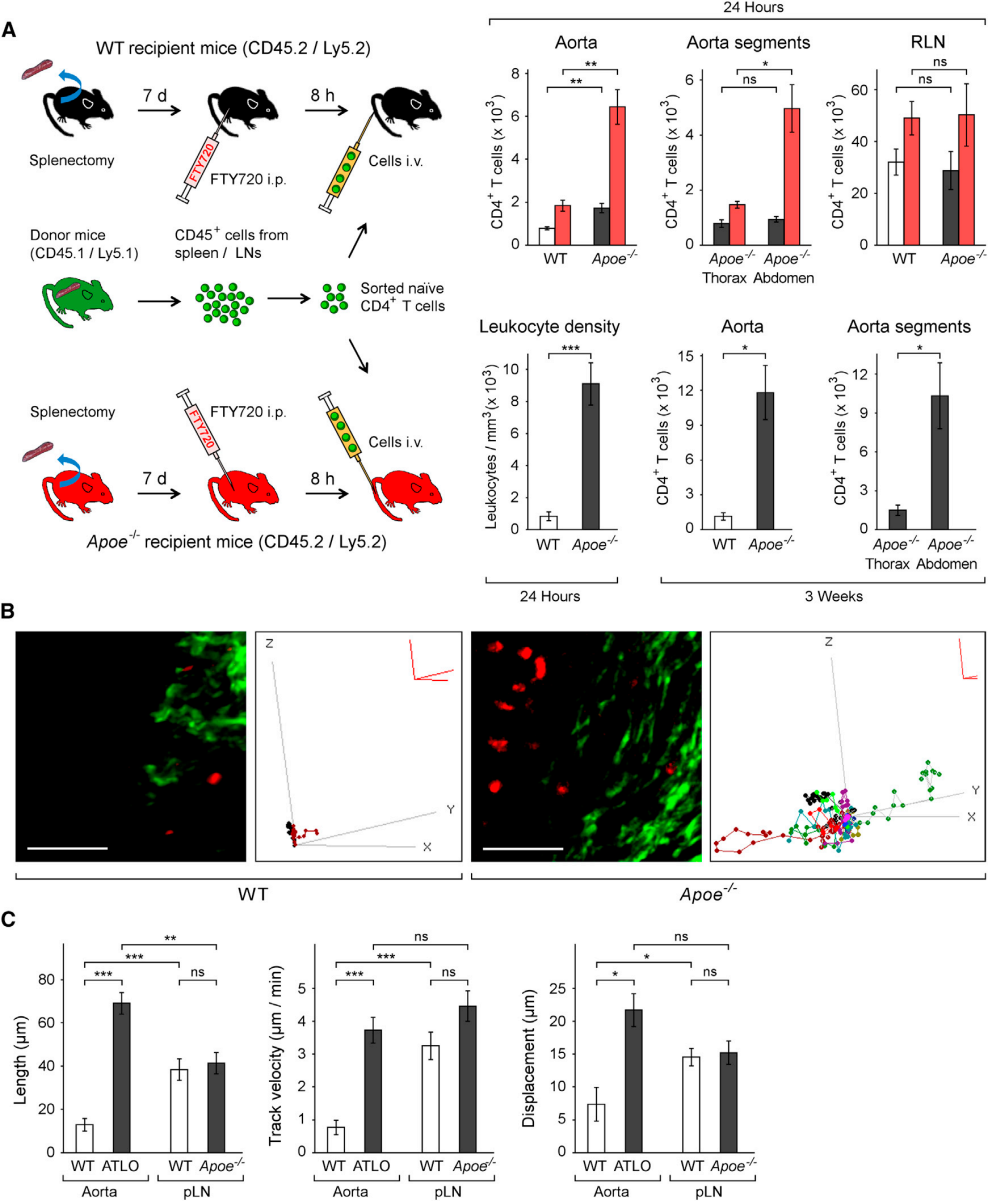
ATLOs Recruit Naive CD4^+^ T Cells into the Diseased Arterial Wall and Alter Lymphocyte Motility (A) Recruitment of naive CD4^+^ T cells. Experimental approach with 78- to 85-week-old recipient and 9- to 12-week-old donor mice. Ly5.1 naive CD4^+^ (CD4^+^CD62L^+^CD69^−^CD25^−^CD44^−^) T cells were analyzed at 24 hr (upper three right panels) or after 3 weeks (lower two right panels) in total aortas (upper left, red columns), aorta segments (upper middle, red columns), and/or RLNs (upper right, red columns). Control mice were not splenectomized or FTY720-treated (WT open columns; *Apoe*^−/−^ mice black columns). Means ± SEM of upper three and lower two right panels (n = 3 experiments with one mouse per genotype per experiment) were determined by two-sided Student’s t test. ^∗^p ≤ 0.05, ^∗∗^p ≤ 0.01, ^∗∗∗^p ≤ 0.001. Leukocyte density in *Apoe*^−/−^ (n = 8 mice) or WT (n = 11 mice) abdominal adventitiae was determined by MPLSM 24 hr after i.v. injection of CMTPX-labeled leukocytes (lower left panel). A two-tailed Wilcoxon-Mann-Whitney test was applied on mouse means. (B) Leukocyte movement. 3D plots of leukocyte movement in ATLOs or WT adventitiae were generated from MPLSM by placing the starting point of each track at the origin of the axes (Movies S1 and S2). Scale bars represent 80 μm; Scale red axis represents 10 μm. (C) Leukocyte motility. Parameters: length, track velocity, and displacement were determined by MPLSM in 78- to 85-week-old *Apoe*^−/−^ (n = 8 mice) or WT (n = 11 mice) abdominal aorta adventitiae or in *Apoe*^*−/−*^ (n = 9 mice) or WT (n = 9 mice) popliteal LNs (pLNs) as described in the Experimental Procedures. Two-tailed Wilcoxon-Mann-Whitney test corrected for multiple testing (Bonferroni) was performed on mouse means. ^∗^p ≤ 0.05, ^∗∗^p ≤ 0.01, ^∗∗∗^p ≤ 0.001.

Recruitment of naive T cells in SLO-sufficient mice has not been studied in any TLO (Woodland and Kohlmeier, 2009). To demonstrate naive T cell recruitment by ATLOs, we had to consider two issues: naive T cells egress SLOs via efferent lymph vessel sphingosine 1-phosphate receptor type 1 (S1PR1), and the recirculation rate of naive T cells in SLOs is constitutively high. Consequently, the presence of T_EM_ or T_CM_ cells in SLOs or ATLOs cannot be taken as evidence for their local generation (Schwab and Cyster, 2007). Hence, demonstration of participation of ATLOs in naive T cell recruitment in atherosclerosis requires that T cell recirculation had to be prevented. Application of the S1PR1 antagonist FTY720 in spleen-sufficient mice led to a reduction of blood T cells by >95% and increased numbers in SLOs, indicating that T cell recirculation was largely but not entirely prevented. As FTY720 is less effective in preventing spleen T cell egress, splenectomy was required in addition to FTY720 treatment. Thus, a combined splenectomy and FTY720-treatment approach was adopted (Matloubian et al., 2004). In splenectomized and FTY720-treated mice, the total number of recruited naive CD4^+^ T cells was higher in *Apoe*^*−/−*^ adventitiae, ATLOs, and in *Apoe*^−/−^ and WT RLNs when compared to their untreated counterparts (Figure 3A). Furthermore, *Apoe*^*−/−*^ aortas recruited >3 times more cells when compared to WT aortas and *Apoe*^*−/−*^ abdominal aorta recruited markedly more naive CD4^+^ T cells when compared to the thoracic aorta (Figure 3A). These data indicated that naive CD4^+^ T cell recirculation in both *Apoe*^−/−^ and WT adventitiae is at least in part regulated by egress via S1PR1. However, *Apoe*^*−/−*^ and WT RLNs recruited similar numbers of naive CD4^+^ cells within 24 hr (Figure 3A). We observed that *S1pr1* mRNA expression was >40% lower in ATLOs when compared to ATLO-free abdominal aorta adventitia in the transcriptome atlas (data not shown). For naive CD8^+^ T cells, similar data were obtained (data not shown). When leukocyte recruitment was determined using multiphoton laser-scanning microscopy (MPLSM), ATLOs showed 10-fold homing rates when compared to WT aortas at 24 hr (Figure 3A). The first stages of naive T cell priming in SLOs, i.e., immigration and activation (see below), occur within 24 hr (Mempel et al., 2004, Miller et al., 2004). To examine the long-term fate of naive CD4^+^ T cells, we transferred flow cytometry-purified Ly5.1 T cells into aged Ly5.2 *Apoe*^*−/−*^ or WT mice and aortas and SLOs were analyzed after 3 weeks. *Apoe*^*−/−*^ aortas retained >10-fold more T cells versus WT aortas (Figure 3A, lower right panels). These data indicated that ATLOs have a large capacity to recruit naive T cells into the arterial wall and generate T_EM_ cells (Figure 3A). In support of clonal expansion of ATLO T cells, we noticed large numbers of CD3^+^Ki67^+^ T cells (data not shown). *Apoe*^*−/−*^ RLNs and spleen also showed higher numbers of transferred naive CD4^+^ T cells after 3 weeks when compared to those in WT mice consistent with the possibility that ATLOs mediate priming and generation of T_EM_ cells followed by their subsequent migration into SLOs (data not shown).

### ATLO T Cells Acquire Movement Parameters Resembling Those in LNs

Naive T cell priming in SLOs (Miller et al., 2004, Schneider et al., 2006) requires specific migration characteristics in T cell areas to allow extended T cell-DC interactions. We used MPLSM (Maffia et al., 2007) to compare T cell movement in ATLOs with those in the WT adventitia and LNs (Miller et al., 2004). Adoptively transferred CMTPX-labeled leukocytes were examined at 24 hr. Numerous cells were visible in ATLOs though few cells were detectable in WT adventitia (Figure 3B). Cell movement was greatly enhanced in ATLOs (Figure 3B), whereas cells in WT aortas were nearly motionless with cell movement confined to a radius of ∼10 μm (Figures 3B and 3C; Movie S1). In contrast, cells moved rapidly in ATLOs and cells that migrated >100 μm were observed in each of 8 *Apoe*^*−/−*^ mice (Figure 3B right panel; Figure 3C left panel; Movie S2). In addition to increased track velocity (3.7-fold increase, p < 0.001; Figure 3C middle panel), cells in ATLOs showed increased displacement (2.6-fold, p < 0.05; Figure 3C right panel) typical for the behavior of naive T cells in LNs (Zinselmeyer et al., 2005). We directly compared movement in *Apoe*^*−/−*^ versus WT peripheral LNs that do not drain the atherosclerotic aorta, i.e., popliteal LNs (pLNs). However, movement parameters in *Apoe*^*−/−*^ and WT pLNs were identical (Figure 3C, right panel).

### ATLOs Activate and Convert Naive CD4^+^ T Cells into Induced Treg Cells

**Figure 4:**
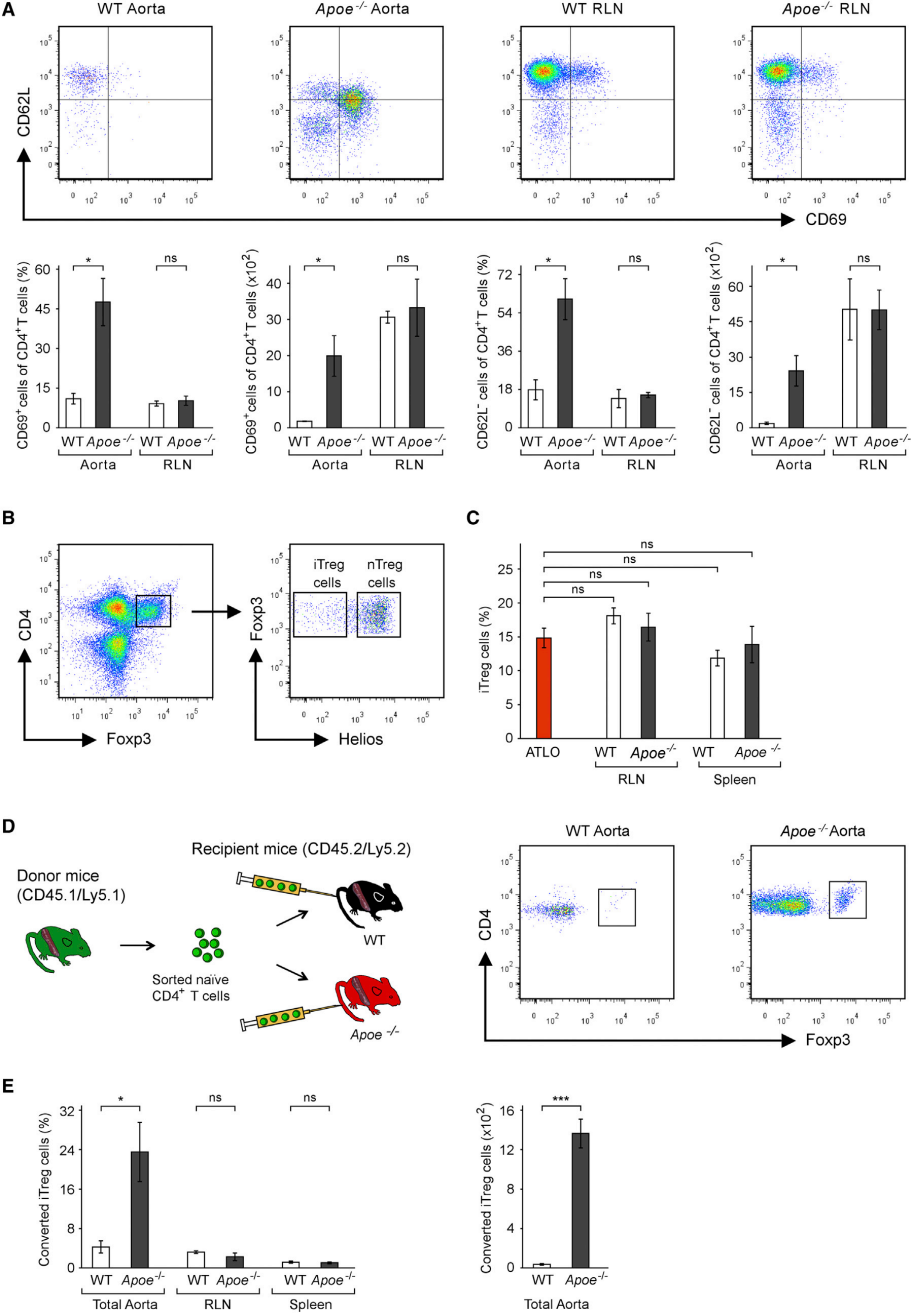
ATLOs Prime Naive CD4^+^ T Cells and Convert Some of Them into iTreg Cells (A) Activation of naive CD4^+^ T cells in situ. 78- to 85-week-old *Apoe*^*−/−*^ or WT mice were splenectomized and FTY720-treated as described in Figure 3A. Ly5.1 cells were analyzed by flow cytometry at 24 hr in total aortas or RLNs for CD62L and CD69 expression (upper panel), and their absolute numbers and frequencies among recruited cells (lower panel) (see also Figures S5). (B) nTregs and iTregs in ATLOs. CD4^+^Foxp3^+^ Treg cells in ATLOs were analyzed for Helios expression. (C) Frequencies of iTreg cells in total CD4^+^Foxp3^+^ Treg cells. (D) Conversion of naive CD4^+^ cells into iTregs. Experimental approach to determine naive CD4^+^ (CD4^+^CD62L^+^CD69^−^CD25^−^CD44^−^) T cell conversion into iTreg cells (left), and flow cytometry shows the converted Foxp3^+^ iTreg cells from the transfer cell gate after 3 weeks. (E) Quantification of converted iTreg cells from migrated naive CD4^+^ T cells 3 weeks after transfer. Data are representative of three (A, D, and E), or four (B and C) experiments with one mouse per genotype per experiment. Two-tailed Student’s t test for (A) and (E); two-tailed Wilcoxon-Mann-Whitney test corrected for multiple testing (Bonferroni) for (C). ^∗^p ≤ 0.05, ^∗∗^p ≤ 0.01, ^∗∗∗^p ≤ 0.001.

To compare activation of naive T cells in *Apoe*^*−/−*^ and WT aortas and SLOs, we examined naive CD4^+^ T cells for CD62L and CD69 expression at 24 hr in splenectomized and FTY720-treated aged mice (Figure 3A, left panel). WT aortas induced CD69 and downregulated CD62L in ∼10% and ∼17% of TCRβ^+^ Ly5.1 naive CD4^+^ T cells, respectively, whereas ATLOs induced CD69 and downregulated CD62L in ∼47% and∼57% of Ly5.1 naive CD4^+^ T cells, respectively (Figure 4A). No differential naive CD4^+^ T cell activation by *Apoe*^*−/−*^ versus WT RLNs was observed (Figure 4A). Likewise, naive CD8^+^ T cells were selectively recruited, activated, and educated by ATLOs in *Apoe*^−/−^ mice but not by WT aortas, or WT and *Apoe*^−/−^ RLNs (Figure S5). We examined whether Treg cells were similarly activated by ATLOs using GFP-Treg cells purified from spleens and LNs of transgenic *Foxp3-DTR-GFP* mice (Kim et al., 2007). However, we failed to observe a similar activation by ATLOs: Foxp3^+^-GFP cells remained CD103^lo^PD-1^lo^CD25^+^CD62L^+^ even 3 weeks after transfer contrasting to their endogenous Treg cell counterparts (Figure 2D; data not shown). These data suggested that the majority of aorta-educated endogenous ATLO Treg cells (Figure 2D) might result from clonal selection whose steady-state homeostasis from adoptively transferred cells might not be achievable within 3 weeks. We next reasoned that ATLOs might participate in peripheral conversion of naive CD4^+^ T cells into induced Helios^−^ iTreg cells known to restrict the effector function of T_EM_ cells with high efficiency (Bilate and Lafaille, 2012). We first analyzed endogenous ATLO Treg cells for Helios expression. ∼18% of all ATLO Treg cells were Foxp3^+^Helios^−^ iTreg cells (Figures 4B and 4C). To examine peripheral conversion of naive CD4^+^ T cells into iTreg cells, we transferred Ly5.1 naive CD4^+^ T cells into recipient mice and analyzed their corresponding aortas and SLOs after 24 hr or 3 weeks. No iTreg cell generation could be observed within 24 hr. However, after 3 weeks, *Apoe*^*−/−*^ aortas had converted ∼30% whereas WT aortas had converted only ∼5% of the migrated naive CD4^+^ T cells into iTreg cells (Figures 4D and 4E). WT RLNs and spleens showed low naive CD4^+^ T cell conversion into iTreg cells (Figure 4E). The absolute number of iTreg cells was 38-fold higher in *Apoe*^−/−^ when compared to WT aortas (Figure 4E).

### ATLOs Show an Aberrant Composition of Antigen-Presenting Cells

**Figure 5:**
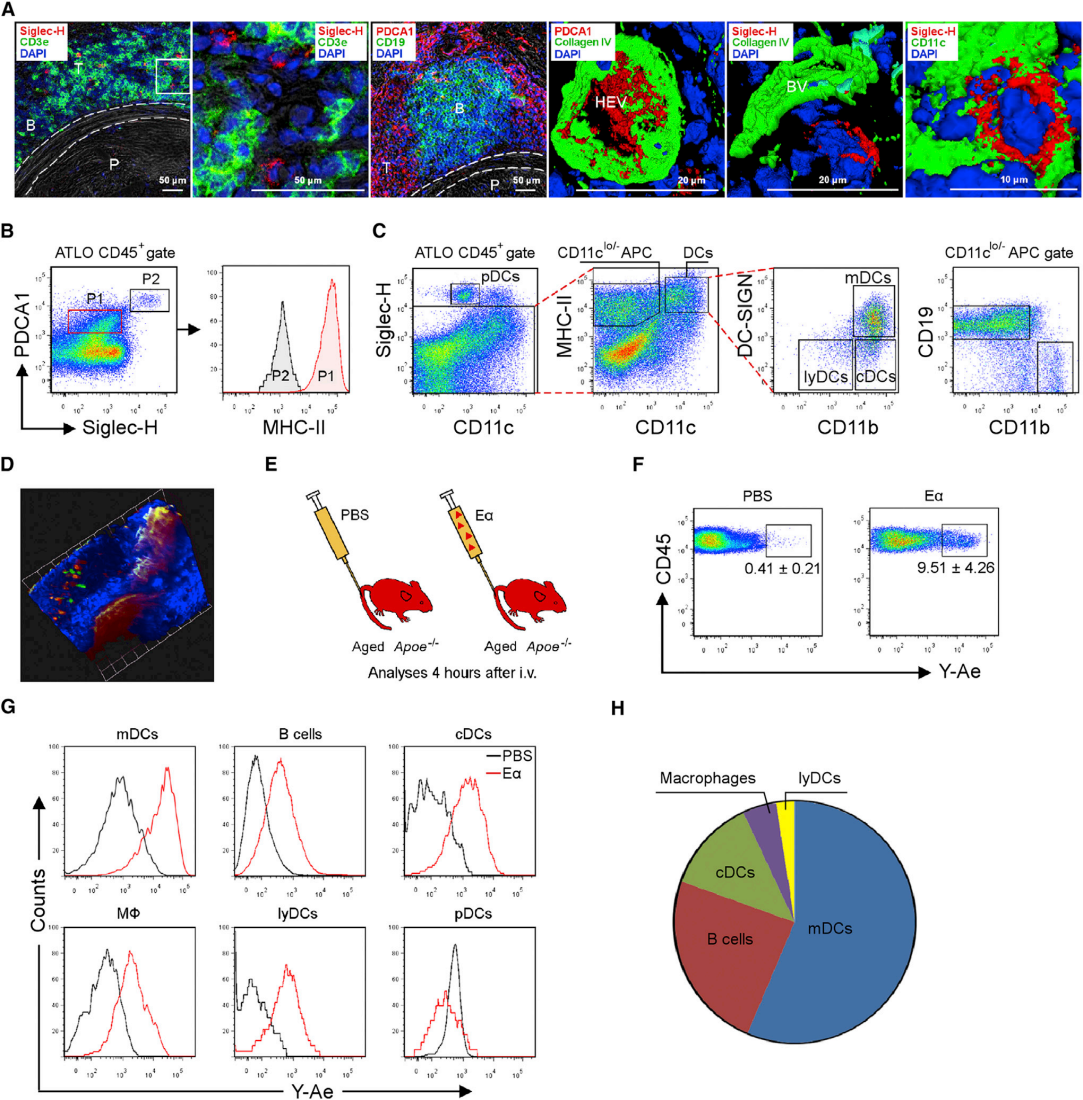
ATLOs Present Antigen by an Unusual Set of APCs (A) PDCA1^+^ and Siglec-H^+^ cells in ATLOs. Immunofluorescence staining show preferential location of Siglec-H^+^ pDCs in CD3^+^ T cell area (T) of ATLO and at higher magnification of boxed area (first two panels), the staining of PDCA1^+^ cells in ATLO T cell area and 3D-reconstructed colocalization of PDCA1 with Collagen IV in HEV endothelial cells (second two panels), and 3D-reconstructed co-staining of Siglec-H with Collagen IV in ATLO blood vessels (BV) and CD11c (third two panels; n = 3 mice). (B) pDCs are MHC-II^lo^. Flow cytometry analyses show co-staining of PDCA1 and Siglec-H on ATLO CD45^+^ cells (left) and MHC-II expression on PDCA1^lo^Siglec-H^−^ cells (P1) and PDCA1^hi^Siglec-H^+^ cells (P2)(right). (C) Gating strategy for APCs. Flow cytometry plots show the gating strategy for APC subtypes in pre-gated CD45^+^ cells from plaque-removed abdominal aorta. (D) OT-II T cells-ATLO cDC interactions. 3D image of ATLO OT-II T cell-DC interactions in situ (n = 8 mice). Grid Unit = 42.6 μm. Projection: Lumen toward adventitia. T cells are red, DCs are green (see also Movies S3 and S4). (E) Approach for Eα or PBS injection. (F) Y-Ae^+^ cells in ATLOs. Flow cytometry plots indicate Y-Ae^+^ cells among CD45^+^ cells from plaque-removed ATLO-bearing abdominal aorta segments as in C. (G) Y-Ae^+^ APC subtypes. Histograms show comparisons of Y-Ae expression in mDCs, B cells, cDCs, macrophages, lyDCs and pDCs from PBS- or Eα-GFP-injected mice. (H) Composition of ATLO APCs. Pie chart depicts the composition of Eα-presenting APCs in ATLOs (recipient mice 78- to 85 weeks old, donor OT-II mice 9- to 12 weeks old). Flow cytometry data are representative of four experiments with one mouse per genotype per experiment (B, C, and E–H).

Intima DCs expand during atherogenesis in young cholesterol-fed *Ldlr*^*−/−*^ mice (Choi et al., 2011, Weber et al., 2011). We had observed earlier that ATLOs (Gräbner et al., 2009, Moos et al., 2005, Zhao et al., 2004) contain cDCs, macrophages, and B cells. Here, we characterized the lineages and functional activities of the major ATLO APCs. Staining of ATLOs for PDCA1 and Siglec-H showed that PDCA1^+^ was widely expressed, whereas Siglec-H was expressed only on few cells, i.e., plasmacytoid DCs (pDCs) (Figures 5A and 5B). To avoid contamination of ATLO DCs by previously described intima DCs (Choi et al., 2011), we removed atherosclerotic plaques before aorta cell suspensions were prepared. PDCA1^+^Siglec-H^+^ pDCs were major histocompatibility complex molecule class II low (MHC-II^lo^) (Figure 5B, P2), and ∼1%–2% CD45^+^ cells were CD11c^lo^Siglec-H^+^ pDCs. ∼80% of the CD11c^hi^MHC-II^+^ APCs were CD11b^+^DC-SIGN^+^ mDCs (myeloid DCs), ∼15% CD11b^+^DC-SIGN^−^ cDCs (conventional DCs), and ∼5% CD11b^−^DC-SIGN^−^ lyDCs (lymphoid DCs), while the MHC-II^+^CD11c^lo/−^ APCs harbored ∼80% CD19^+^CD11b^−^ B cells, and ∼10% CD19^−^CD11b^+^ macrophages (Figure 5C).

### ATLO APCs Present Exogenous Antigen In Vivo

Two models of in vivo antigen presentation were established. At 24 hr after transfer of transgenic OT-II CD4^+^ T cells followed by OVA or PBS intravenous (i.v.) administration, ATLOs of aged *Apoe*^*−/−*^*CD11c-YFP* mice were examined (Figure 5D). ATLOs in *Apoe*^*−/−*^*CD11c-YFP* mice showed long-lasting clustering of OT-II CD4^+^ T cells with YFP^+^ DCs confirming and extending previous observations obtained by others in in vitro added T cells (Koltsova et al., 2012) (Figure 5D; Movies S3 and S4). To determine which APC type(s) present antigen in ATLOs, we employed the Eα/Y-Ae system (Itano et al., 2003) using the model antigen Eα-GFP together with the Y-Ae monoclonal antibody recognizing the Eα peptide in the context of MHC-II (I-A^b^). Aged *Apoe*^−/−^ mice received either Eα-GFP or PBS i.v., and presentation of antigen was assessed by flow cytometry (Figures 5E–5G) (Macritchie et al., 2012). All Y-Ae^+^ cells in the abdominal aorta were MHC-II^hi^ (data not shown). Prototypic DC subtype markers showed that ∼55% of MHC-II^hi^Y-Ae^+^ APCs were CD11c^+^CD11b^+^DC-SIGN^+^ mDCs, ∼24% were CD11c^-/lo^CD11b^−^CD19^+^ B cells, 12% CD11c^+^CD11b^+^DC-SIGN^−^ cDCs, ∼4% CD11c^−/lo^CD11b^+^CD19^−^ macrophages, and ∼2% CD11c^+^CD11b^−^DC-SIGN^−^ lyDCs (Figure 5H). To study the origin of ATLO mDCs, we transferred flow cytometry-purified Ly5.1 bone marrow monocytes (CD115^+^CD11c^lo^CD11b^+^F4/80^+^Ly6C^+^Ly6G^lo^PDCA1^−^) to aged *Apoe*^*−/−*^ mice. Donor bone marrow monocytes in spleen and RLNs of *Apoe*^*−/−*^ recipients were CD11c^−^PDCA1^−^, whereas they were CD11c^+^PDCA1^+^CD11b^+^F4/80^+^ in ATLOs after 1 week (data not shown). These data indicated that bone marrow monocytes migrate into ATLOs and locally differentiate into mDCs (Cheong et al., 2010). PDCA1^+^Siglec-H^+^ pDCs represented a minor fraction in ATLOs though none of them were Y-Ae^+^ consistent with low expression of MHC-II (Figure 5B). In summary, these results suggest that mDCs followed by B cells, macrophages, cDCs, and lyDCs are the major ATLO APCs.

### The Immune System of VSMC-*Ltbr*^*−/−*^ Mice Is Indistinguishable from that of WT Mice

**Figure 6:**
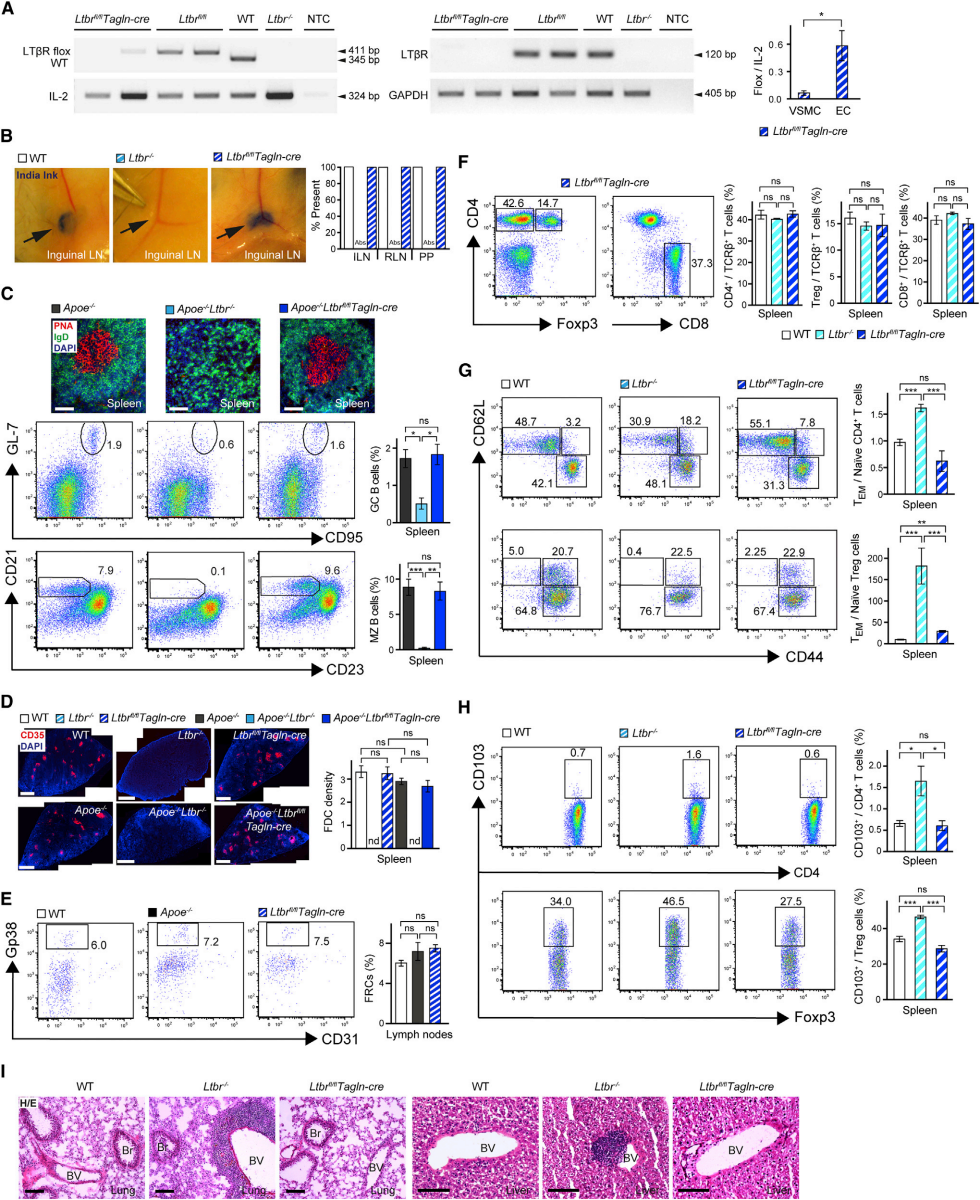
The Immune System of VSMC-LTβR Deficient Mice (A) Ltbr deletion in VSMCs. Validation of genomic *Ltbr* deletion in freshly isolated aortic VSMCs by PCR (left) and of *Ltbr* mRNA (middle); qRT-PCR show reduction of floxed *Ltbr* sequences in cultured aortic VSMCs compared to aortic endothelial cells (ECs) (n = 3 experiments) of *Ltbr*^fl/fl^*Tagln-cre* mice (right); NTC, no template control. (B) SLO neogenesis. Inguinal LNs (ILNs) were visualized by India ink injection into footpads of 8- to 10-week-old mice (n = 8) (left panels, arrow); all other LNs (data not shown) show similar results. SLOs in WT, *Ltbr*^*−/−*^, and *Ltbr*^fl/fl^*Tagln-cre* mice (n = 8) (right); RLN, renal LN; PP, Peyer’s patches; Abs, absent. (C) GC and MZ B cells in spleen. PNA^+^ GC B cells and IgD^+^ follicular MZ B cells in spleen (upper panel) (n = 8–12 sections in 5–7 mice per genotype). Flow cytometry show GC (middle panel) and MZ B cells (lower panel) in B220^+^ B cells from 78- to 80-week-old mice. (D) FDCs in spleen. Representative lower magnification montages of CD35^+^ FDCs and quantification of FDCs in spleen (n = 8–12 sections in 5–7 mice per genotype). (E) FRCs in LNs. Flow cytometry of Gp38^+^CD31^−^ FRCs of CD45^−^Ter119^−^ cells in LNs of 78- to 90-week-old mice (left panel). (F) T cell subsets in spleen. Flow cytometry of CD4^+^Foxp3^−^ T cells, CD4^+^Foxp3^+^ Treg cells, and CD8^+^ T cells of TCRβ^+^ cells of 78- to 90-week-old *Ltbr*^fl/fl^*Tagln-cre* mice. (G) T_EM_ and naive T cells in spleen. Flow cytometry of CD62L^+^CD44^−^ naive, CD62L^−^CD44^+^ T_EM_, and CD62L^+^CD44^+^ T_CM_ cells of CD4^+^ T cells of 78- to 90-week-old mice; T_EM_ per naive T cell ratios of CD4^+^ T cells (upper panel) or CD4^+^ Treg cells (lower panel). (H) CD103^+^ cells in spleen. Flow cytometry plots show CD103^+^ cells from CD4^+^ (upper panel) or Treg (lower panel) cell gate of 78- to 90-week-old mice; bar graphs show the percentage comparison of CD103^+^ cells among CD4^+^ T or Treg cells at right. (I) Perivascular infiltrates in peripheral tissues. Hematoxylin and eosin (H/E) staining show leukocyte infiltrates around blood vessels (BVs) in lungs and liver. n = 3 mice per genotype. Br, bronchiole. Scale bar represents 50 μm (C and I); 500 μm (D). Flow cytometry data are representative of three experiments with pooled one to two mice per genotype per experiment (C and E–H). Data represent means ± SEM; p values were determined by unpaired Student’s t test or by multiple testing (Bonferroni) using the GEE model as described in the Experimental Procedures. ^∗^p < 0.05; ^∗∗^p < 0.01; ^∗∗∗^p < 0.001; ns, not significant (see also Figure S6).

The role of TLOs in unresolvable inflammatory diseases remains unknown. Experimental approaches to unambiguously address this issue have not emerged (Aloisi and Pujol-Borrell, 2006, Mohanta et al., 2014, Pitzalis et al., 2014, Weyand et al., 2001). To make attempts to overcome this major obstacle, we took advantage of previous observations in aged *Apoe*^*−/−*^ mice: The lymphorganogenic chemokines, i.e., CXCL13 and CCL21, are selectively expressed in VSMCs that are sandwiched between atherosclerotic plaques and ATLOs of aged *Apoe*^*−/−*^ mice; agonistic LTβR antibodies trigger CXCL13 expression in WT VSMCs, but not in *Ltbr*^*−/−*^ VSMCs, in vitro(Lötzer et al., 2010); and antagonistic LTβR antibodies disrupt ATLO structure and attenuate aorta *Cxcl13* mRNA in vivo(Gräbner et al., 2009). These data raised the important possibility that VSMC-LTβRs might participate in ATLO neogenesis as lymphoid tissue organizer cells. Furthermore, we speculated that if ATLOs were to be impacted by VSMC-LTβRs, then they might also affect atherosclerosis. Following these lines of thought, we reasoned that selective blockade of the putative LTβR signaling pathway in VSMCs should interfere with ATLO neogenesis without affecting the immune system systemically. To evaluate this hypothesis, we generated *Apoe*^−/−^*Ltbr*^fl/fl^*Tagln-cre* mice to delete the LTβR selectively in VSMCs (Boucher et al., 2003, Lepore et al., 2005) (Figure 6A). Because controls for hyperlipidemic mice that lack LTβRs systemically and show multiple defects of their immune system (Fütterer et al., 1998, Stopfer et al., 2004), we also generated *Apoe*^*−/−*^*Ltbr*^*−/−*^ mice (Figure 6A). Both *Apoe*^−/−^*Ltbr*^fl/fl^*Tagln-cre* and *Apoe*^*−/−*^*Ltbr*^*−/−*^ mice showed concentrations of plasma lipids that were identical to those of their *Apoe*^*−/−*^ counterparts (Figure S6A). We next studied the immune systems of WT, *Apoe*^*−/−*^, their control brethren, i.e., *Ltbr*^*−/−*^ and *Apoe*^*−/−*^*Ltbr*^*−/−*^ mice, and *Ltbr*^fl/fl^*Tagln-cre* and *Apoe*^*−/−*^*Ltbr*^fl/fl^*Tagln-cre* mice. Young and aged *Apoe*^*−/−*^*Ltbr*^*−/−*^ or *Ltbr*^*−/−*^ mice lacked LNs and Peyer’s patches (Figure 6B, data not shown). We observed disruption of spleen structure, including a lack of germinal center (GC) B and marginal zone (MZ) B cells (Figure 6C), of spleen follicular dendritic cells (FDCs) (Figure 6D), of spleen marginal metallophilic macrophages (Figure S6B), and blood vessels of spleen white pulp (Figure S6B) though numbers of LN fibroblastic reticular cells (FRCs) (Figure 6E) and the composition of T cell subsets in spleen were comparable to those of WT mice (Figure 6F). However, compared to aged WT mice, *Ltbr*^*−/−*^ mice showed increased ratios of T_EM_/naive splenic and blood T cell subsets (Figures 6G and S6C), higher numbers of splenic CD103^+^CD4^+^ T cells (Figure 6H), lower numbers of splenic CD8^+^CD103^+^ T cells (Figure S6D), higher numbers of CD4^+^CD103^+^ T cells in blood, and lower numbers of CD103^+^ Treg cells in blood (Figure S6D), lower or equal numbers of CD4^+^PD-1^+^, PD-1^+^Treg T cell subsets, and increased numbers of splenic and blood CD8^+^PD-1^+^ T cells (Figures S6E and S6F), slightly lower numbers of DCs (not significant due to high variability) and higher numbers of splenic macrophages (Figure S6G), and leukocyte infiltrates in nonlymphoid tissues (Figure 6I). In contrast, young and aged *Apoe*^*−/−*^*Ltbr*^fl/fl^*Tagln-cre* or *Ltbr*^fl/fl^*Tagln-cre* mice did not show comparable changes of these abnormalities (Figures 6 and S6). These data provide evidence that *Apoe*^*−/−*^*Ltbr*^fl/fl^*Tagln-cre* mice do not show major systemic alterations of their immune system.

### VSMC-LTβRs Maintain ATLO Structure and Protect against Atherosclerosis

**Figure 7:**
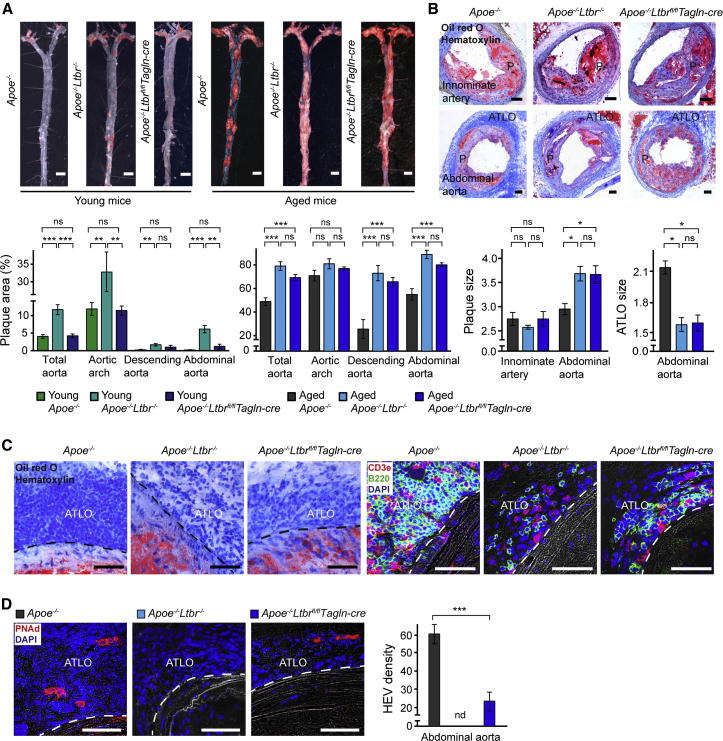
VSMC-LTβRs Protect against Atherosclerosis (A and B) Atherosclerotic plaque and ATLO sizes. Sudan-IV-stained aortas of young (32–35 weeks; n = 3) and aged (78- to 85 weeks old; n = 4–6) mice (A, upper panel), and Oil red O/hematoxylin stained innominate arteries (n = 3–8) and abdominal aortas (n = 4–8) (B, upper panel). Atherosclerotic lesions in different parts of the aorta were quantified as percentage of plaque areas (n = 3–6 mice per genotype (A, lower panels); plaque (P) sizes and ATLO sizes in different aorta segments were quantified as percentage of plaque areas, intima/media, and ATLO/media ratios, respectively (n = 5–10 sections in 3–8 mice per genotype) (B, lower panels). (C and D) Effect of Ltbr deletion on the ATLO structure. Histological and immunofluorescence stainings show ATLO cellularity (C) and HEV abundance (D) in abdominal aorta segments (n = 5–8 sections in n = 3–4 mice per genotype). Scale bar represents 2.5 mm (A); 100 μm (B); and 50 μm (C and D). Data represent means ± SEM; p values were determined by two-tailed Student’s t test or by multiple testing (Bonferroni) using the GEE model as described in the Supplemental Experimental Procedures. ^∗^p < 0.05; ^∗∗^p < 0.01; ^∗∗∗^p < 0.001; ns, not significant, p > 0.05; nd, not detectable.

Both 32- to 35-week-old and 78- to 85-week-old *Apoe*^*−/−*^*Ltbr*^*−/−*^ mice revealed markedly accelerated atherosclerosis as evidenced by increased en face lipid staining and intima media ratios (Figures 7A and 7B). However, 32- to 35-week-old *Apoe*^*−/−*^*Ltbr*^fl/fl^*Tagln-cre* mice did not show augmented atherosclerosis (Figure 7A), indicating that the VSMC-LTβR does not affect the early stages of the disease. However, both aged *Apoe*^*−/−*^*Ltbr*^*−/−*^ and aged *Apoe*^*−/−*^*Ltbr*^fl/fl^*Tagln-*cre mice showed aberrant ATLO structures as revealed by the reduced size of ATLOs, loose T and B cell infiltrates, loss of separate T and B cell areas, and a complete absence or a markedly reduced number of high endothelial venules (HEVs) in ATLOs, respectively (Figures 7B–7D). However, aged *Apoe*^−/−^*Ltbr*^fl/fl^*Tagln-cre* mice revealed robust acceleration of atherosclerosis whose magnitude was indistinguishable from that of age-matched *Apoe*^*−/−*^*Ltbr*^*−/−*^ mice (Figure 7A) and this acceleration was greater in the abdominal aorta when compared to other parts of the arterial tree (Figure 7B). These data indicate that VSMC-LTβRs maintain ATLO structure and cellularity and protect against atherosclerosis in a site-specific and age-dependent way.

## Discussion

This study has identified ATLOs as the principal lymphoid tissue that controls atherosclerosis T cell responses during aging and suggests that VSMC-LTβRs protect against atherosclerosis by maintaining ATLO structure and cellularity.

ATLO activities are selective and robust involving major steps of an antigen-specific primary T cell response: Recruitment of naive T cells, modulation of T cell motility toward characteristics of those in SLOs, activation of naive CD4^+^ and naive CD8^+^ T cells, antigen presentation, generation of CD4^+^, CD8^+^, and Treg memory cells, education of T_EM_ and T_CM_ cells, and conversion of naive CD4^+^ T cells into iTreg cells. Thus, the immune response in atherosclerosis is carried out in the adventitia during aging though it is assumed to be organized in plaques and/or in SLOs in young mice. Together with the systemic age-associated changes of all T cell compartments, these data suggest the paradigm that the senescent immune system is capable of selectively employing a TLO to organize unresolvable disease-specific immune responses.

A fundamental unanswered question in the immunology of unresolving inflammation relates to the impacts of TLOs in any disease setting. Although there is correlative evidence that TLOs can afford disease protection in certain acute pathogen-triggered diseases and cancers (Aloisi and Pujol-Borrell, 2006, Drayton et al., 2006, Mohanta et al., 2014, Moyron-Quiroz et al., 2004, Pitzalis et al., 2014, Roozendaal and Mebius, 2011), circumstantial evidence including clinical association studies has led investigators to assume that TLOs enforce rather than attenuate chronic inflammation and—in particular—autoimmune diseases (Aloisi and Pujol-Borrell, 2006, Pitzalis et al., 2014, Weyand et al., 2001). Direct evidence for this proposition, however, would require blockade of TLO function without affecting the immune system systemically. This has not been a viable option in the past because molecules that specifically regulate TLO neogenesis in adult organisms as opposed to those that regulate SLO formation during ontogeny have not been identified (Drayton et al., 2006, Fütterer et al., 1998, Roozendaal and Mebius, 2011). Yet, data reported here show that the structure and cellularity of a TLO can be altered without affecting SLOs. This was possible by targeting the LTβR of VSMCs using the late differentiation marker of these cells, i.e., SM22α/Transgelin (Boucher et al., 2003). Together with the evidence that *Apoe*^*−/−*^*Ltbr*^fl/fl^*Tagln-cre* or *Ltbr*^fl/fl^*Tagln-cre* mice lacked changes of SLO structure and cellularity when compared to SLOs of *Apoe*^*−/−*^*Ltbr*^*−/−*^ or *Ltbr*^*−/−*^ mice but developed major alterations of ATLOs indicate that TLOs in other peripheral inflammatory and autoimmune diseases could also be targeted. Distinct immune cells (i.e., the lymphoid tissue inducer cells), are known to interact with LTβRs on stromal mesenchymal cells, i.e., the stromal organizer cells that give rise to fibroblastic reticular cells (FRCs) in adult SLOs, to form SLOs during ontogeny (Roozendaal and Mebius, 2011). The observation that *Apoe*^*−/−*^*Ltbr*^fl/fl^*Tagln-*cre mice showed disruption of ATLO structure and size supports the view that VSMCs can adopt lymphoid tissue organizer-like characteristics in advanced atherosclerosis.

Regarding the possible protection from atherosclerosis by ATLOs in aged mice, several aspects of our data merit attention: ATLOs generate both pro-inflammatory T_EM_ and T_CM_ cells and anti-inflammatory nTreg and iTreg cells (Ait-Oufella et al., 2006). However, immunosuppressive leukocytes appear to restrict their effector counterparts under the pathogen-free conditions used in this study. Possibly, the highly activated ATLO nTreg and iTreg cells shift the balance between pro-atherogenic and anti-atherogenic T cell subtypes toward inhibition of immune responses by restricting activation in and the release of T_EM_ and T_CM_ cells from ATLOs. It will be a challenge for future studies to identify the specific roles of ATLO iTreg cells on atherosclerosis. Moreover, B cell subsets in ATLOs remain to be characterized and could also impact the balance of pro- versus anti-atherogenic lymphocytes. However, this favorable fine-tuning of pro- versus anti-inflammatory lymphocytes could well change under different conditions. Thus, systemic infections by pathogens, including those that lead to activation of Toll-like receptors, are known to activate DCs and antigen-specific T_EM_ and T_CM_ cells during bouts of exacerbations in multiple sclerosis and rheumatoid arthritis (Aloisi and Pujol-Borrell, 2006, Lopez-Diego and Weiner, 2008), and similar events might occur in atherosclerosis.

In summary, these data define interactions between the aging/senescent immune system, the media of aged arteries, and hyperlipidemia and characterize the role of abdominal aorta segments in generating highly territorialized ATLOs. ATLOs appear to function not only as powerhouses of advanced atherosclerosis immunity but also seemingly afford strong protection from advanced atherosclerosis in an age- and site-specific way. These data raise the important possibility that TLOs in other forms of unresolvable inflammation also provide immunoprotection. Further studies are needed to identify mechanisms by which ATLO APCs and immune cell subtypes protect from atherosclerosis. Future studies should also help to isolate putative (auto)immune T and B cells and to uncover modes of peripheral tolerance breakdown during clinical stages of advanced atherosclerosis. This might facilitate identification of mechanisms underlying the poorly understood phenomenon of acute exacerbations and relapses in atherosclerosis and autoimmune diseases. A better understanding of ATLO immunity might thus be of major clinical significance as TLO-directed therapies are being evaluated for the treatment of chronic inflammatory diseases, autoimmune diseases, and cancer (Aloisi and Pujol-Borrell, 2006, Mohanta et al., 2014, Pitzalis et al., 2014, Weyand et al., 2001).

## Experimental Procedures

### Mice

C57BL/6J WT and *Apoe*^*−/−*^ mice were housed in the animal facility of Jena University. *Ltbr*^*−/−*^ and *Ltbr*^fl/fl^ mice were provided by Yang-Xin Fu, University of Chicago. Tg (Tagln-cre) 1Her/J mice were backcrossed onto the C57BL/6 background for 5 generations using speed congenics. *Ltbr*^fl/fl^*Tagln-cre* mice were generated by crossing *Ltbr*^fl/fl^ mice with *Tagln-cre* mice. *Apoe*^*−/−*^*Ltbr*^*−/−*^ and *Apoe*^*−/−*^*Ltbr*^fl/fl^*Tagln-cre* mice were generated by crossing *Apoe*^*−/−*^ mice with *Ltbr*^*−/−*^ or *Ltbr*^fl/fl^*Tagln-cre* mice. CD45.1/Ly5.1 and *Relb*^*−/−*^ mice were bred at the Leibniz-Institute for Age Research Jena. *Foxp3-DTR-GFP* mice were provided by Alexander Rudensky. *Apoe*^*−/−*^*CD11c-YFP* and *OT-II* mice were bred at the Research Facility at the University of Glasgow. Mice were fed a standard rodent chow and kept under pathogen-free conditions. Animal procedures were conducted according to guidelines of the local Animal Use and Care Committees and the National Animal Welfare Laws.

### Preparation of Single Cell Suspensions from Aorta, Spleen, LN, and Blood

Cell suspensions from aorta was prepared by enzyme digestion as previously described with minor modifications and detailed in the Supplemental Experimental Procedures (Gräbner et al., 2009).

### Cell Purification and Adoptive Transfers

Lymphocytes from SLOs were isolated from donor mice and injected into recipient mice, whereas for MPLSM studies, leukocyte cell suspensions and transgenic CD4^+^ T cells were prepared from SLOs of WT and OT-II mice, respectively, and i.v. injected as described in the Supplemental Experimental Procedures.

### Flow Cytometry

Cells for flow cytometry were pretreated with purified anti-mouse CD16/32 mAb to block Fc receptors as described (Gräbner et al., 2009). Cells were incubated with Abs for 25 min at 4°C, washed twice, and, when required, incubated with secondary mAbs or streptavidin conjugates for 20 min. After washing, 8-color flow cytometry measurements were performed on a FACSCanto II™ (BD Bioscience), and data were analyzed using FlowJo (Tree Star). Antibodies are described in the Supplemental Experimental Procedures.

### Histology, Immunofluorescence, and Morphometry

Tissues were prepared and stored as described (Gräbner et al., 2009). 10 μm cross-sections were prepared and every 10^th^ serial section at 100 μm intervals was stained with Oil Red O/hematoxylin to delineate ATLOs. Immunofluorescence staining was performed as previously described (Gräbner et al., 2009), using marker antibodies as described in online methods. DAPI was used to stain DNA. Secondary antibodies were used as previously described (Zhao et al., 2004). For 3D imaging, z stacks were prepared at 0.3 μm intervals using a Plan Apochromatic 63× differential interference contrast (DIC) oil objective (NA1.4) with a scan zoom factor 3.1 and then processed with Zen 2009 Light Edition (Zeiss) and further processed as described in the Supplemental Experimental Procedures.

### Atherosclerotic Lesion Analyses

Aortas were prepared, stained with Sudan-IV for en face atherosclerosis analysis, and the extent of atherosclerosis was assessed in total aorta, aortic arch, descending aorta, and abdominal aorta using ImageJ software as described (Cao et al., 2009, Schmitt et al., 2014). The extent of atherosclerosis was assessed in total aorta, aortic arch, descending aorta, and abdominal aorta using ImageJ software as described in the Supplemental Experimental Procedures. In addition, plaque size and corresponding ATLO size were quantified in Oil red O/hematoxylin stained serial sections of the innominate artery and the abdominal aorta below the renal artery at 100 μm intervals as described (Gräbner et al., 2009).

### Cell Culture

Aortic VSMCs and ECs were harvested from aortas of 10- to 12-week-old mice as described (Gräbner et al., 2009) and detailed in the Supplemental Experimental Procedures.

### Administration of Eα Antigen

To study the ability of APCs to present systemic antigen, the Eα-GFP/Y-Ae system was used as previously described (Itano et al., 2003). Briefly, aged *Apoe*^*−/−*^ mice were i.v. injected either with 1 mg of Eα antigen or PBS, and assays were performed 4 hr later.

### LCM and Generation of Microarrays

LCM and microarray analyses were performed as previously reported (Gräbner et al., 2009; C.Y. and A.J.R.H., unpublished data).

### Statistical Analyses

To compare flow cytometry data or morphometry data of multiple mouse groups, we used the generalized estimating equation model (GEE) as described in the Supplemental Experimental Procedures.

## Author Contributions

D.H. designed and performed experiments and wrote the manuscript; S.K.M. designed and performed experiments and wrote the manuscript; C.Y., L.P., Z.M., P.S., G.G., N.M., G.D., P.G., F.L.B., A.I., S.R.S., T.L., D.T., L.H., M.B., and R.G. performed experiments; C.W. wrote the manuscript; I.B.M., J.M.B., and P.G. designed the experiments; P.M. designed and performed experiments and wrote the manuscript; F.W. designed experiments and wrote the manuscript; A.J.R.H. designed experiments and wrote the manuscript.

## Accession Numbers

The Gene Expression Omnibus (GEO) accession number for the microarray data reported in this paper is GSE40156.

## Supplemental Information

Document S1. Figures S1–S6, Supplemental Experimental Procedures

Document S2. Transcript Profiles of WT and *Apoe*^–/–^ Aorta, Spleen, Blood, and Renal Lymph Node, Related to Figures 1, S1, S2, and S3

Movie S1. Imaging of CMPTX-Labeled Cells in the Aged WT Aorta Adventitia, Related to Figure 3

Movie S2. Imaging of CMPTX-Labeled Cells in ATLO of aged *Apoe*^*−/−*^ Abdominal Aorta Adventitia, Related to Figure 3

Movie S3. Imaging of Antigen-Specific T-DC Clustering in ATLOs, Related to Figure 5

Movie S4. High-Magnification Imaging of Antigen-Specific T-DC Clustering in ATLOs, Related to Figure 5

Document S3. Article plus Supplemental Information
