# Supplementary material for: Artery Tertiary Lymphoid Organs Control Aorta Immunity and Protect against Atherosclerosis via Vascular Smooth Muscle Cell Lymphotoxin β Receptors
Source: Immunity. 2015 Jun 16;42(6):1100–15. doi: 10.1016/j.immuni.2015.05.015 (PMC4678289; doi:10.1016/j.immuni.2015.05.015)
Supplement: Document S1. Figures S1–S6, Supplemental Experimental Procedures [file mmc1.pdf]

Immunity

Supplemental Information

Artery Tertiary Lymphoid Organs Control Aorta Immunity and Protect against Atherosclerosis via Vascular Smooth Muscle Cell Lymphotoxin β Receptors

Desheng Hu, Sarajo K. Mohanta, Changjun Yin, Li Peng, Zhe Ma, Prasad Srikakulapu, Gianluca Grassia, Neil MacRitchie, Gary Dever, Peter Gordon, Francis L. Burton, Armando Ialenti, Suleman R. Sabir, Iain B. McInnes, James M. Brewer, Paul Garside, Christian Weber, Thomas Lehmann, Daniel Teupser, Livia Habenicht, Michael Beer, Rolf Grabner, Pasquale Maffia, Falk Weih, and Andreas J.R. Habenicht

SUPPLEMENTAL INFORMATION

Figures S1 - S6

Movies S1 - S4

Supplemental Experimental Procedures

Supplemental References

SUPPLEMENTAL FIGURES

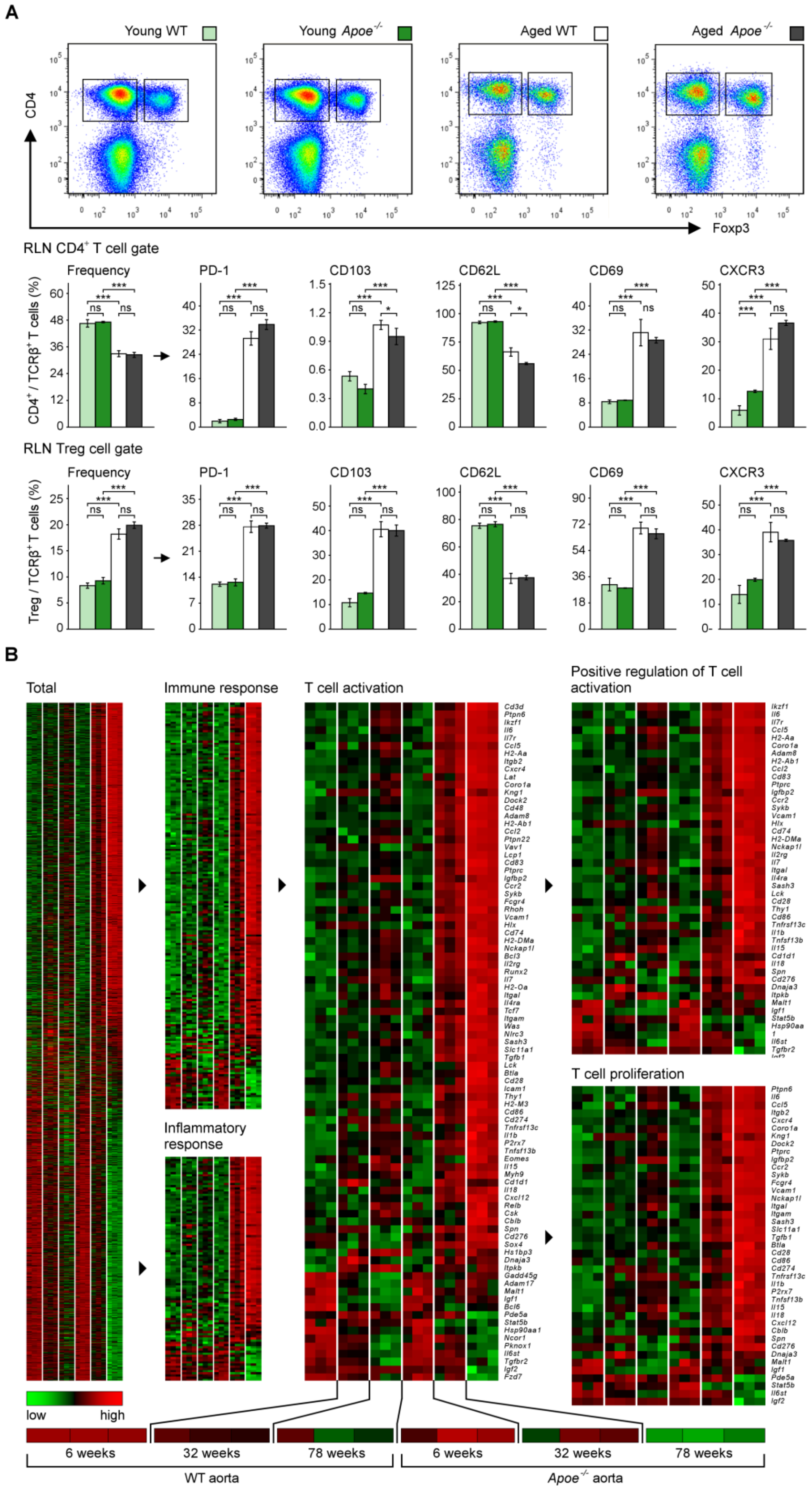

Figure S1, related to Figure 1. Age-associated changes in T cell subtype composition and activation in RLNs and aortas of WT versus *Apoe*$^{-/-}$ mice.

(A) Age-associated changes in CD4^{+} T cells and activation in RLNs. Flow cytometry analyses for PD-1, CD103, CD62L, CD69, and CXCR3 of RLN CD4^{+} T and Treg cells in 9-12 weeks young WT (light green) or *Apoe*$^{-/-}$ (dark green) and 78-85 weeks old WT (white) or *Apoe*$^{-/-}$ mice (black). Data are representative of three independent experiments with pooled 1-2 mice per genotype per experiment with two technical replicates. Means ± SEM and *P* values were determined by multiple testing (Bonferroni) using the GEE model as described in online Methods. * $P \leq 0.05$; ** $P \leq 0.01$; *** $P \leq 0.001$. (B) Age-associated mRNA profiles in WT and *Apoe*$^{-/-}$ aortas. Transcriptomes of WT or *Apoe*$^{-/-}$ aortas of 6, 32, and 78 weeks old mice (*n*=3 mice per genotype per time point). Differentially expressed total numbers of genes (left) or transcripts for GO terms immune response, inflammatory response, T cell activation, positive regulation of T cell activation, and T cell proliferation are displayed as heatmaps. Absolute numbers of signal intensities are reported in Table S1A1. ANOVA with Benjamini-Hochberg correction was applied.

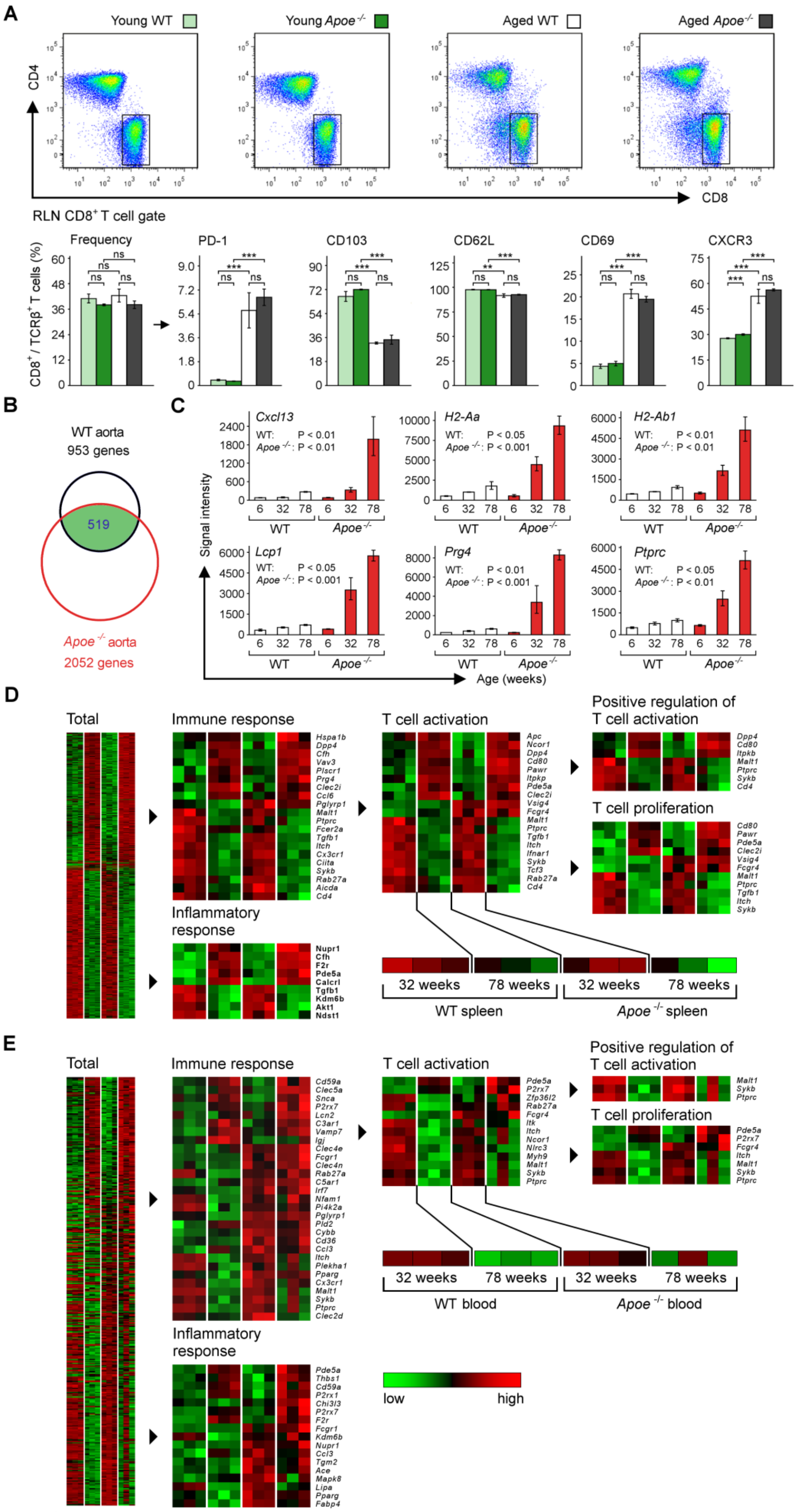

A
Young WT
Young Apoe-/-
Aged WT
Aged Apoe-/-
CD4
CD8
RLN CD8+ T cell gate
Frequency
PD-1
CD103
CD62L
CD69
CXCR3
CD8+ / TCRβ+ T cells (%)
ns
***
**
B
WT aorta
953 genes
519
Apoe-/- aorta
2052 genes
C
Cxcl13
H2-Aa
H2-Ab1
Lcp1
Prg4
Ptprc
WT: P < 0.01
Apoe-/-: P < 0.01
WT: P < 0.05
Apoe-/-: P < 0.001
WT: P < 0.01
Apoe-/-: P < 0.01
WT: P < 0.05
Apoe-/-: P < 0.001
WT: P < 0.01
Apoe-/-: P < 0.001
WT: P < 0.05
Apoe-/-: P < 0.01
Signal intensity
Age (weeks)
WT
Apoe-/-
D
Total
Immune response
T cell activation
Positive regulation of T cell activation
T cell proliferation
Inflammatory response
Hspa1b Dpp4 Cfh Vav3 Plscr1 Prg4 Clec2i Ccl6 Pglyrp1 Malt1 Ptprc Fcer2a Tgfb1 Itch Cx3cr1 Ciita Sykb Rab27a Aicda Cd4
Apc Ncor1 Dpp4 Cd80 Pawr Itpkb Pde5a Clec2i Vsig4 Fcgr4 Malt1 Ptprc Tgfb1 Itch Ifnar1 Sykb Tcf3 Rab27a Cd4
Dpp4 Cd80 Itpkb Malt1 Ptprc Sykb Cd4
Cd80 Pawr Pde5a Clec2i Vsig4 Fcgr4 Malt1 Ptprc Tgfb1 Itch Sykb
Nupr1 Cfh F2r Pde5a Calcrl Tgfb1 Kdm6b Akt1 Ndst1
32 weeks
78 weeks
WT spleen
Apoe-/- spleen
E
Total
Immune response
T cell activation
Positive regulation of T cell activation
T cell proliferation
Inflammatory response
Cd59a Clec5a Snca P2rx7 Lcn2 C3ar1 Vamp7 Igj Clec4e Fcgr1 Clec4n Rab27a C5ar1 Irf7 Nfam1 Pi4k2a Pglyrp1 Pld2 Cybb Cd36 Ccl3 Itch Plekha1 Pparg Cx3cr1 Malt1 Sykb Ptprc Clec2d
Pde5a P2rx7 Zfp36l2 Rab27a Fcgr4 Itk Itch Ncor1 Nlrc3 Myh9 Malt1 Sykb Ptprc
Malt1 Sykb Ptprc
Pde5a P2rx7 Fcgr4 Itch Malt1 Sykb Ptprc
Pde5a Thbs1 Cd59a P2rx1 Chi3l3 P2rx7 F2r Fcgr1 Kdm6b Nupr1 Ccl3 Tgm2 Ace Mapk8 Lipa Pparg Fabp4
32 weeks
78 weeks
WT blood
Apoe-/- blood
low
high

Figure S2, related to Figures 1, 2. Age-associated changes in genes and transcript profiles.

(A) Age-associated changes in $CD8^+$ T cells and activation in RLNs. As in Figure S1, $CD8^+$ T cells were analyzed. Data are representative of three independent experiments with pooled 1-2 mice per genotype per experiment with two technical replicates. Means ± SEM and *P* values were determined by multiple testing (Bonferroni) using the GEE model as described in online Methods. * $P \leq 0.05$; ** $P \leq 0.01$; *** $P \leq 0.001$. (B) Venn diagram of differentially regulated genes during aging in WT or $Apoe^{-/-}$ mice. Microarray analyses of total aorta RNA of 6, 32, and 78 weeks old WT or *Apoe*$^{-/-}$ mice (3 mice per genotype per age group). (C) Differential age-associated and genotype-associated gene expression of selected genes in WT versus *Apoe*$^{-/-}$ total aorta transcriptomes. Means ± SEM (3 mice per genotype per age group). ANOVA with Benjamini-Hochberg correction was applied. (D-E) Age-associated transcript profiles of WT *and Apoe*$^{-/-}$ spleen (D) or blood (E) of 32 and 78 weeks old mice (3 mice per genotype per age group). Differentially expressed total numbers of genes (left) and transcripts in GO terms immune response, inflammatory response, T cell activation, positive regulation of T cell activation, and T cell proliferation are displayed as heatmaps. Absolute numbers of signal intensities are reported in Table S1A2, A3. ANOVA with Benjamini-Hochberg correction was applied.

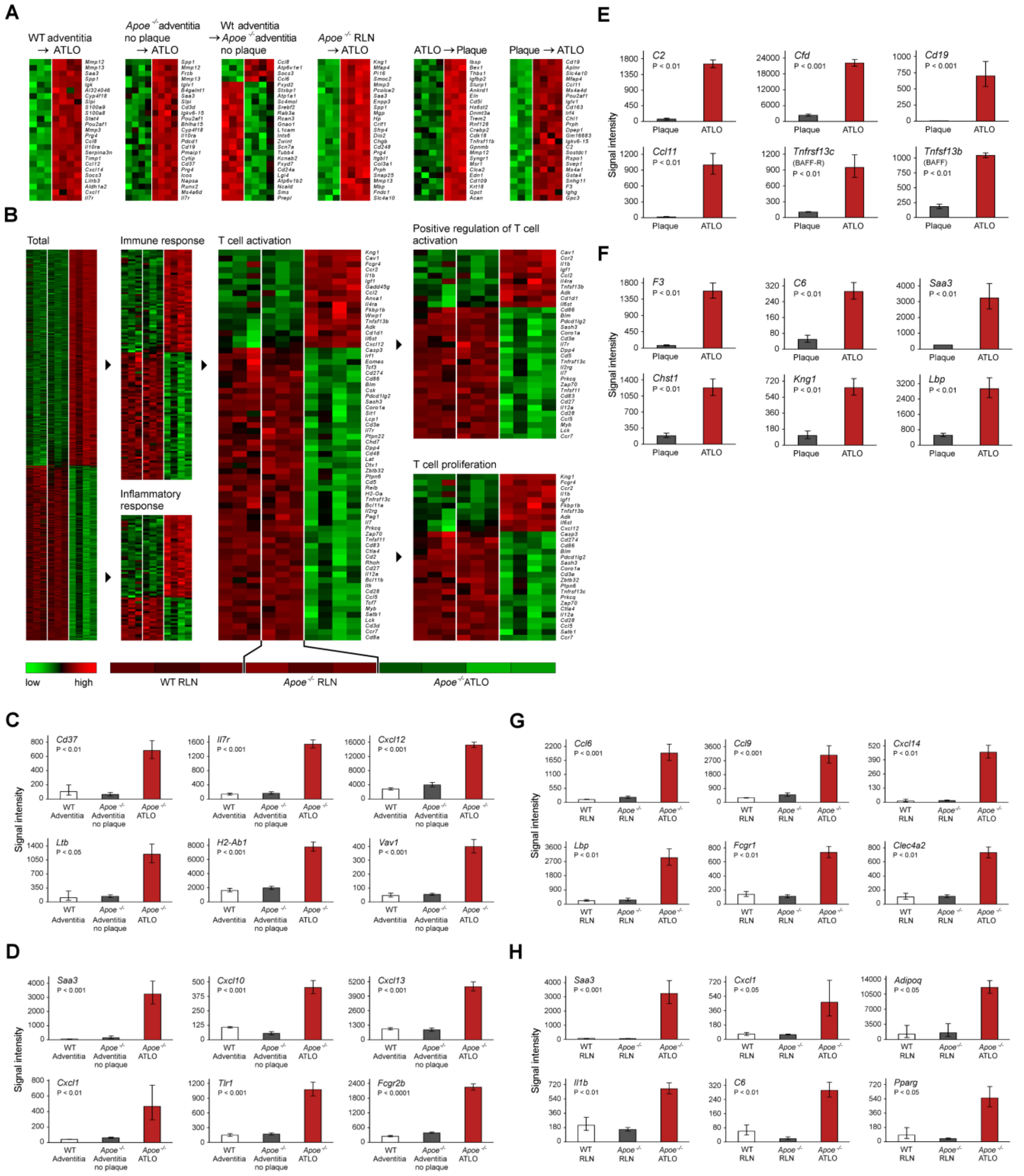

Figure S3, related to Figure 1. Comparisons of transcriptome maps.

(A) Top 20 differentially expressed transcripts in two tissue comparisons of transcript maps of 78 weeks old WT adventitia and RLNs, ATLOs, *Apoe*$^{-/-}$ adventitia no plaque, *Apoe*$^{-/-}$ RLN,

and plaque. (B) Lymph node cluster in three tissue comparisons of WT and *Apoe*$^{-/-}$ RLNs versus ATLOs. 78 weeks old WT RLN, *Apoe*$^{-/-}$ RLN, and ATLO transcripts are shown as heatmaps of total differentially expressed genes (left) and mRNAs in respective GO terms (right). 3 WT and 4 *Apoe*$^{-/-}$ mice were used for microarray. Absolute numbers of signal intensities are reported in Table S1B. ANOVA with Benjamini-Hochberg correction was applied. (C-F) Comparison of signal intensities of selected genes from of 78 weeks old mice are shown in different clusters: (F) adventitia cluster-Immune response; (D) adventitia cluster-Inflammatory response; (E) plaque-ATLO cluster- Immune response; (F) plaque-ATLO cluster- Inflammatory response. Means ± SEM (n=3 WT mice, n=4 *Apoe*$^{-/-}$ mice), ANOVA with Benjamini-Hochberg correction (C-D) or two-sided Student's t-test (E-F) were applied. (G-H) LCM-derived 78 weeks old WT RLNs, 78 weeks old *Apoe*$^{-/-}$ RLNs, and ATLOs were examined. Comparison of signal intensities of selected genes are shown in (G) LN cluster-Immune response; (H) LN cluster-Inflammatory response. Means ± SEM (n=3 WT, n=4 *Apoe*$^{-/-}$ mice). ANOVA with Benjamini-Hochberg correction was applied.

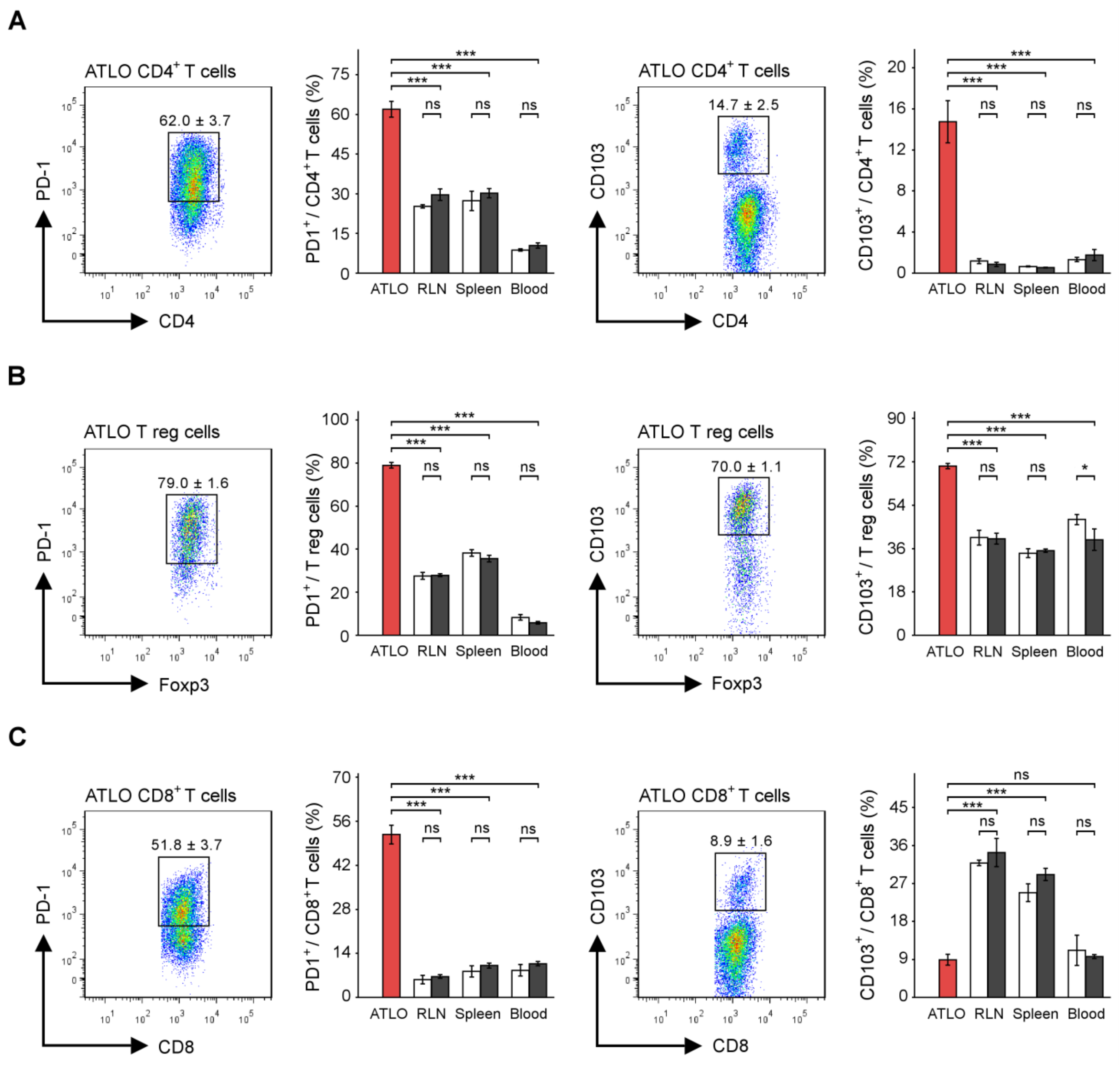

Figure S4, related to Figure 2. T cell education in ATLOs versus SLOs and blood T cell subsets.

(A) $CD4^+$ T cell education; (B) Treg cell education; (C) $CD8^+$ T cell education from ATLO (red), RLN, spleen, and blood of 78-85 weeks old WT (white) or *Apoe*$^{-/-}$ (black) mice. Flow cytometry plots show the expression of PD-1 or CD103 on ATLO $CD4^+$ T cells, Treg cells, and $CD8^+$ T cells (far left and right panels), and their percentages were compared with those in RLN, spleen, and blood (left and far right panels), respectively. Data are

representative of four independent experiments with pooled 1-2 mice per genotype per experiment. Means, SEM, and P values corrected for multiple testing (Bonferroni) were estimated using the GEE model. * $P \leqslant 0.05$; ** $P \leqslant 0.01$; *** $P \leqslant 0.001$.

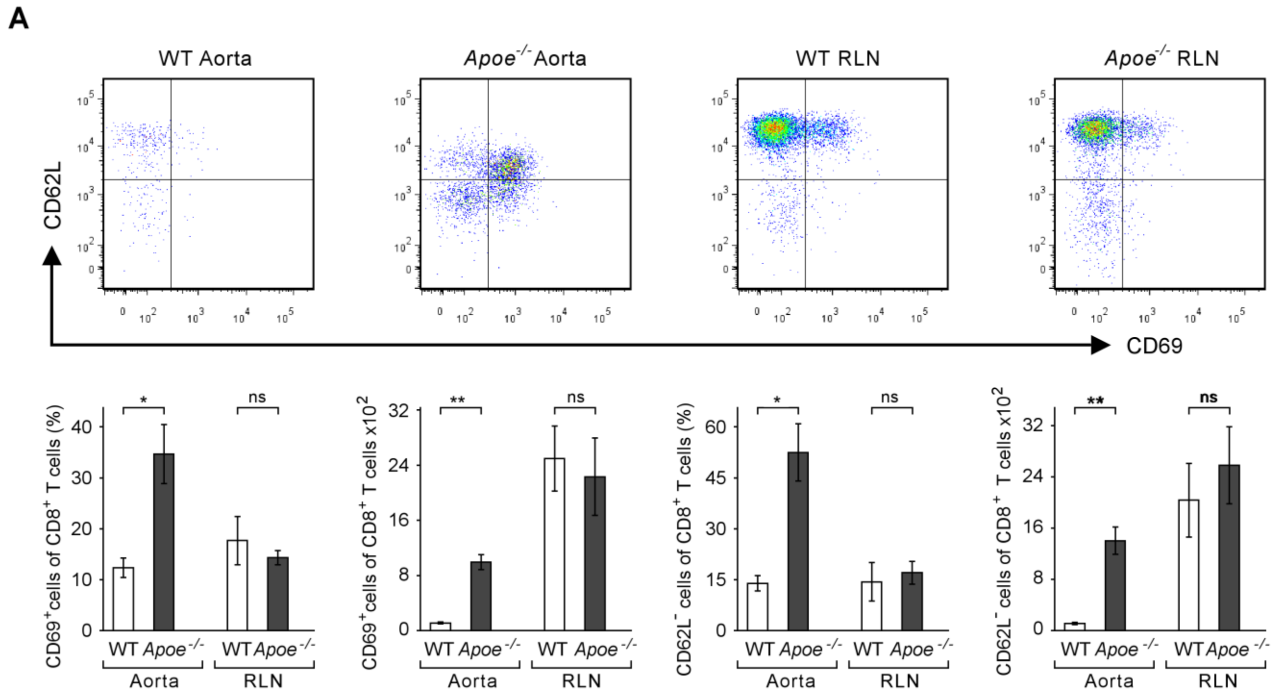

Figure S5, related to Figure 4. Rapid activation of naïve CD8$^+$ T cells by ATLOs *in situ*.

Activation of naïve CD8$^+$ T cells in ATLOs. 78-85 weeks old WT and *Apoe*$^{-/-}$ mice were splenectomized and treated with FTY720 as described in the main manuscript. 20 x 10^6 flow cytometry-purified TCRβ$^+$CD69$^-$CD62L$^+$CD44$^-$ donor T cells were injected i.v. and recruited CD8$^+$ Ly5.1 cells were analyzed after 24 h in total aortas or RLNs for CD69 and CD62L expression. First left and first right panels (lower row) show the percentages of CD69$^+$ or CD62L$^-$ T cells of all recruited CD8$^+$ Ly5.1 T cells in WT or *Apoe*$^{-/-}$ aortas or RLNs, respectively. Second left and second right panels show absolute numbers of CD69$^+$ or CD62L$^-$ T cells of all recruited CD8$^+$ Ly5.1 T cells in WT or *Apoe*$^{-/-}$ aortas or RLNs, respectively. Three independent experiments with one mouse per genotype per experiment were done. Two-sided Student's t-test was applied; means, SEM, and P values were determined. * $P \leq 0.05$, ** $P \leq 0.01$, *** $P \leq 0.001$.

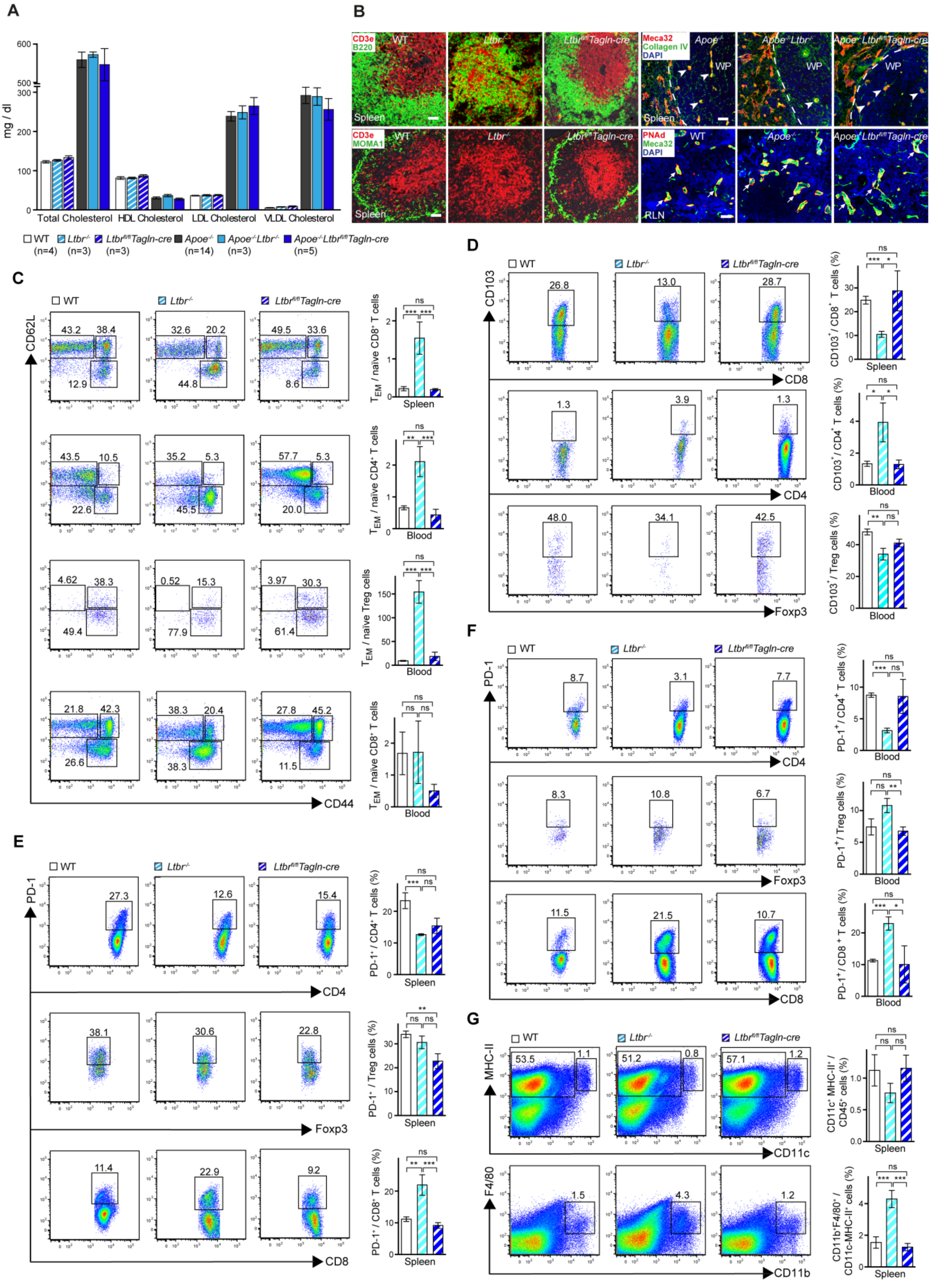

A
mg / dl
Total Cholesterol
HDL Cholesterol
LDL Cholesterol
VLDL Cholesterol
WT (n=4)
Ltbr-/- (n=3)
Ltbrfl/flTagln-cre (n=3)
Apoe-/- (n=14)
Apoe-/-Ltbr-/- (n=3)
Apoe-/-Ltbrfl/flTagln-cre (n=5)
B
CD3e
B220
MOMA1
Spleen
WT
Ltbr-/-
Ltbrfl/flTagln-cre
Meca32
Collagen IV
DAPI
PNAd
Apoe-/-
Apoe-/-Ltbr-/-
Apoe-/-Ltbrfl/flTagln-cre
WP
RLN
C
CD62L
CD44
TEM / naïve CD8+ T cells
TEM / naïve CD4+ T cells
TEM / naïve Treg cells
Spleen
Blood
D
CD103
CD8
CD4
Foxp3
CD103+ / CD8+ T cells (%)
CD103+ / CD4+ T cells (%)
CD103+ / Treg cells (%)
E
PD-1
PD-1+ / CD4+ T cells (%)
PD-1+ / Treg cells (%)
PD-1+ / CD8+ T cells (%)
F
G
MHC-II
CD11c
F4/80
CD11b
CD11c+ MHC-II+ / CD45+ cells (%)
CD11b+F4/80+ / CD11c-MHC-II+ cells (%)

Figure S6, related to Figure 6. The immune system of *Apoe*$^{-/-}$*Ltbr*$^{fl/fl}$*Tagln-cre* mice

(A) Serum cholesterol in 78-82 weeks old mice of different genotypes (n= 3-14 mice per genotype group). (B) Spleen and LN architecture was analyzed by examining CD3^{+} T cells, B220^{+} B cells, MOMA1^{+} MZ metallophilic macrophages (left panels), and collagen IV^{+}Meca32^{+} blood vessels (arrow heads) in white pulp (WP, dotted line) (upper right panels), and PNAd^{+}Meca32^{+} HEVs (arrows). (n=8-12 sections in 5-7 mice per genotype). Scale 50 µm. (C) T_{EM}, naïve cells in spleen and blood. Flow cytometry of CD62L^{+}CD44^{-} naïve T cells, CD62L^{-}CD44^{+} T_{EM} cells, and CD62L^{+}CD44^{+} T_{CM} cells in 78-90 weeks old mice; T_{EM}/naïve cell ratio of spleen CD8^{+} T (upper panel), blood CD4^{+} T (middle upper panel), blood Treg cells (middle lower panels) or blood CD8^{+} T cells (lower panel). (D) CD103^{+} expression on spleen and blood T cells. Flow cytometry of CD103^{+} cells of spleen CD8^{+} T (upper panel), blood CD4^{+} T (middle panel), blood Treg (lower panels) cells of 78-90 weeks old mice; frequencies at right. (E-F) Flow cytometry of PD-1^{+} cells in CD4^{+} T (upper panel), Treg (middle panel), and CD8^{+} T (lower panel) cells of spleen (E) or blood (F) in 78-90 weeks old mice; frequency at right. (G) Flow cytometry of spleen CD11c^{high}MHC-II^{+} cDCs (upper panel) and spleen CD11b^{+}F4/80^{+} macrophages (lower panel) of CD45^{+} cells of 78-90 weeks old mice; frequency at right. Flow cytometry data are representative of four experiments with one mouse per genotype per experiment (C-G). Data represent means ± SEM; P values were determined by two-sided Student's t-test or by multiple testing (Bonferroni) using the GEE model as described in online Methods. * $P < 0.05$; ** $P < 0.01$; *** $P < 0.001$; ns, not significant, $P > 0.05$.

SUPPLEMENTAL MOVIES

Video S1, related to Figure 3. Imaging of CMPTX-labeled cells in the aged WT aorta adventitia

24h after transfer few near stationary leukocytes (red) are visible in the WT adventitia. Elastic fibers (green) in lamina media are evident due to autofluorescence. Video was generated from stacks of 31 planes acquired at 1.2 μm *z*-axis increments using Volocity 5 software. View using Quick Time Player (Video S1).

Video S2, related to Figure 3. Imaging of CMPTX-labeled cells in ATLO of aged *Apoe*$^{-/-}$ abdominal aorta adventitia.

24 h after transfer numerous highly motile leukocytes (red) are visible in ATLO of an aged *Apoe*$^{-/-}$ abdominal aorta segment. Elastic fibers (green) in the lamina media are evident due to autofluorescence. Video was generated from stacks of 21 planes acquired at 1.2 μm *z*-axis increments using Volocity 5 software. View using Quick Time Player (Video S2).

Video S3, related to Figure 5. Imaging of antigen-specific T-DC clustering in ATLOs

24 h after transfer numerous OT-II CD4^{+} T cells (red) and CD11c-YFP^{+} cells (green) are visible in ATLO of an OVA-treated aged *Apoe*$^{-/-}$*CD11c-YFP*$^{+}$ mouse. Collagen fibers (blue) in the adventitia are evident due to second harmonic generation. Several slowly moving OT-II CD4^{+} T cells (red) show long-lasting clustering around YFP^{+} DCs (green). Video was generated using Volocity 5 software. View using Quick Time Player (Video S3).

Video S4, related to Figure 5. High magnification imaging of antigen-specific T-DC clustering in ATLOs.

24 h after transfer two OT-II $CD4^+$ T cells (red) cluster around a single CD11c-YFP^+ cell (green) in ATLO of OVA-treated aged *Apoe*$^{-/-}$*CD11c-YFP*$^+$ mouse (right section of the screen). In the left section of the screen a single static OT-II $CD4^+$ T cell (red) shows long-lasting interactions with a single YFP^+ DC (green). Collagen fibers (blue) are evident due to second harmonic generation. Video was generated using Volocity 5 software. View using Quick Time Player (Video S4).

SUPPLEMENTAL EXPERIMENTAL PROCEDURES

Preparation of single cell suspensions from aorta, spleen, LN, and blood

Cell suspensions from aorta was prepared by enzyme digestion as previously described with minor modifications(Galkina et al., 2006). Briefly, *Apoe*$^{-/-}$ or WT mice were anesthetized and abdominal and thoracic aorta segments were dissected following perfusion of the vasculature with 2 mM EDTA in PBS, PBS, and flow cytometry buffer, respectively. The two segments of the aorta were cut into small pieces and digested separately in 2.5 ml enzyme cocktail containing 450 U/ml collagenase type I, 125 U/ml collagenase type XI, 60 U/ml hyaluronidase type I-s, and 60 U/ml DNase1 in Dulbecco's phosphate buffered saline (DPBS) containing 20 mM HEPES. Digestion was carried out at 37° C for 50 min in a water bath with magnetic rotation. Cell suspensions were filtered through a 70 µm cell strainer and the remainder of the aorta tissue was mashed. The cell suspension was centrifuged and resuspended in flow cytometry buffer. Cell suspension from spleen and LNs were prepared by enzyme digestion as described above. Blood leukocytes were isolated according to standard procedures. For experiments described in Figure 5, aorta plaques were removed under a dissection microscope before single cell suspensions were prepared to remove intima APCs. For cell preparation of fibroblastic reticular cells, LNs were digested with collagenase type IV(1 mg/ml, Sigma) and DNase I (40 µg/ml, Roche) for 30 min at 37 °C.

Cell purification and adoptive transfers

Lymphocytes from spleens and LNs were isolated from 9-12 weeks old Ly5.1 mice or FoxP3-DTR-GFP mice. Cells were incubated with biotin-labeled antibodies against CD19, CD8, CD11b, CD11c, NK1.1 for 20 minutes at 4 °C. After washing, cells were incubated with streptavidin Microbeads (Miltenyi Biotec) according to the manufacturer's instructions for 25 min at 4 °C. After washing, cells were resuspended in 2 mM EDTA-PBS buffer at a cell density of $20x10^6$/ml and filtered through a 40 μm cell strainer. $TCRβ^+$ or $CD4^+$ cells from Ly5.1 mice were further enriched through an autoMACS separator (Miltenyi Biotec). Negative selection-derived cell suspensions were incubated with antibodies against TCRβ, CD4, CD25, CD69, CD62L, and CD44. The $TCRβ^+CD25^-CD69^-CD44^-CD62L^+$ or $TCRβ^+CD4^+CD25^-CD69^-CD44^-CD62L^+$ naïve T cells were sorted using a BD FACS Aria to >97% purity. $20\text{-}25×10^6$ purified T cells were i.v. injected into aged *Apoe*$^{-/-}$ or WT mice for T cell recruitment, T cell activation or Foxp3 conversion analyses. GFP^+ Treg cells from FoxP3-DTR-GFP mice were further enriched through an autoMACS separator. Negative selection-derived cells were then incubated with anti-mouse CD4, and $CD4^+GFP^+$ Treg cells were sorted using a BD FACS Aria. $5\text{-}7×10^6$ sorted Treg cells were i.v. injected into aged *Apoe*$^{-/-}$ mice. For monocyte transfer experiments, bone marrow cells were prepared from femurs of 4-6 weeks old Ly5.1 mice. Monocytes were sorted on a BD FACSAria as $CD115^+CD11c^{lo}CD11b^+Ly6C^+Ly6G^{lo}PDCA1^-$cells. $8 × 10^6$ monocytes were i.v. injected into aged *Apoe*$^{-/-}$ mice.

For adoptive transfer in MPLSM studies, leukocyte cell suspensions were prepared from peripheral and mesenteric LNs of WT mice. For preparation of transgenic $CD4^+$ T cells,

axillary, inguinal, and cervical LNs and spleens from OT-II mice were pooled and forced through Nitex mesh (Cadisch Precision Meshes) using a syringe plunger. Suspensions were washed in RPMI 1640 (GIBCO BRL). $CD4^+$ T cells were purified by negative selection with antibodies against CD8a, CD19, CD45R (B220), CD49b (DX5), CD105, MHC class-II, and Ter-119 ($CD4^+$ T cell isolation kit II, Miltenyi Biotec) over MACS columns (Miltenyi Biotec).

Antibodies for flow cytometry

The mAbs for flow cytometry were: CD45-PE-Cy7(30-F11), CD45.1-PercP-Cy5.5/PE-Cy7(A-20), CD3-FITC(145-2C11), TCRβ-PercP-Cy5.5 (H57-597), TCRδ-Biotin/PE (GL3), CD4-eFluor450 (GK1.5), CD8-PE-Cy7/Biotin (53-6.7), CD44-APC (1M7), CD62L-Biotin/FITC (MEL14), CD69-FITC/ Biotin(H1.2F3), NK1.1-Biotin(PK136), CD25-PE-Cy7(PC61.5), PD-1-FITC/Biotin (RMP1-30), CD103-FITC/APC (2E7), CXCR3-APC (CXCR3-173), CD11b-PercP-Cy5.5/ PE(M1/70), CD11c-APC/eFluor450 (N418), F4/80-PE-Cy7(BM8), CD19-eFluor780(1D3), I-A^b-FITC (AF6-120.1), Ter119-PE-Cy7(TER-119), Gr-1-PE-Cy7(RB6-8C5), CD31-FITC (390), Podoplanin-Biotin(eBio8.1.1), PDCA1-FITC/PE(eBio927), CD23-PercpCy5.5(B3B4), Y-Ae-Biotin (specific for Eα52-68 peptide bound to I-Ab, clone: eBioY-Ae), Streptavidin PE/PE-Cy7/APC-Cy7 (all from eBioscience); CD45-V500(30-F11), CD8-V500 (53-6.7), GL-7-FITC (Gl7), CD95-PE(Jo2), CD21/35-PE(7G6) (from BD Bioscience); Helios-APC(22F6) and Siglec-H-APC(551) (from Biolegend). Intracellular Foxp3 staining

was performed with the anti-mouse Foxp3-PE (FJK-16s) staining kit (eBioscience) according to the manufacturer's instructions.

Immunofluorescence microscopy and morphometry

Tissues were dissected and embedded in Tissue Tec (Sakura Finetek), frozen in chilled isopentane over dry ice, and stored at -80 °C. Immunofluorescence staining was performed as previously described (Grabner et al., 2009), using marker antibodies CD3ε (145-2C11; BD), CD4 (RM4-5; BD), CD8 (YTS105.18; Serotec), Foxp3 (ab54501.100; Abcam), PDCA1 (eBio927; eBioscience), Siglec-H (eBio440c; eBioscience), CD11c (N418; AbDSerotec), CD138 (AF3190; R&D Systems), Collagen IV (2150-1470; AbDSerotec), MECA-32; BD), CD45R/B220 (RA3-6B2; BD), MOMA-1 (AbDSerotec), PNAd (MECA-79; BD), CD35/CD21b (CR1; BD), IgD (11-26c.2a; BD), PNA-FITC (Vector Lab). For negative controls, stainings were performed without primary antibodies. Stained sections were analyzed using a confocal laser scanning microscope (CLSM) 510 META (Carl Zeiss, Germany) or Leica SP5 or DM6000 (Mannheim, Germany). 2-3 images per ATLO were acquired and the number of HEVs per μm^2 ATLO area was quantified in serial abdominal aorta sections (n = 5-8). Montages of 3-5 lower magnification (5x) images were generated and the number of FDC clusters per μm^2 spleen area was quantified in 10-12 serial spleen sections (n = 5-7 mice). All images were saved as TIF files and exported into GNU image manipulation program (GIMP) version 2 or Adobe Illustrator CS6 for figure arrangements.

Visualization of LNs

India ink (100 µl, 1:10 diluted in PBS) was injected into footpads of WT, *Ltbr*$^{-/-}$, *Ltbr*$^{fl/fl}$*Tagln-cre* mice as described (Futterer et al., 1998) and examined for the presence of peripheral LNs including inguinal, mesenteric, axillary, popliteal, cervical, lumbar, renal, and paraaortic LNs after 30 minutes by microscopic examination (n=8 mice of 10-12 wks). Peyer's patches in small intestine were counted after incubating the intestine in 10% acetic acid for 10 minutes.

Cell culture

Aortic VSMCs were harvested from aortas of 10-12 weeks old mice as described(Grabner et al., 2009) after isolation of endothelial cells (ECs) by sequential dissection, and collagenase digestion(Kobayashi et al., 2005). VSMCs and ECs were maintained in VSMC growth medium (Dulbecco's modified Eagle's medium (DMEM)/F12/10% fatal bovine serum (FBS)/ 2mM L-glutamine), and EC growth medium (DMEM/20% FBS/2mM L-glutamine/25mM N-2-hydroxyethylpiperazine-N-2-ethane sulfonic acid (HEPES) at pH 7.0 - 7.6/100 µg/ml heparin/100 µg/ml endothelial cell growth supplement (ECGS)/1x non-essential amino acids/1x sodium pyruvate), respectively, and used at passages 1-3.

PCR analyses

DNAs and RNAs from freshly isolated VSMCs and RNAs from cultured SMCs and ECs were extracted as described(Grabner et al., 2009). Genomic PCR and qRT-PCR analyses were performed using the following primers: LTβR flox: 5'-GACGTCTGCTGCTCCCGCTG

and 5’-GCAAACCGTGTCTTGGCTGC; IL-2: 5’-CTAGGCCACAGAATTGAAAGATCT and 5'-GTAGGTGGAAATTCTAGCATCATCC; LTβR: 5'-TGGTGCTCATCCCTACCTTC-3' and 3'-TCCCAAACTCTCCTCCACAC-5'; GAPDH: 5'-AAGAAGGTGGTGAAGCAGGCAT-3' and 3'-GATGGTATTCAAGAGAGTAGGGA-5'.

Animal preparation for MPLSM

Cells were labeled with CMTPX (Molecular Probes Inc.) and $4x10^6$ total leukocytes were injected i.v.. Transgenic $CD4^+$ T cells were injected i.v. into aged *Apoe*$^{-/-}$*CD11c-YFP* recipients. 20h later, *Apoe*$^{-/-}$*CD11c-YFP* recipients received either 200µg of chicken OVA or PBS i.v. and imaged 4h later. The abdominal aorta and popliteal LN (pLN) were harvested and imaged by MPLSM as previously described(Gibson et al., 2012; Millington et al., 2010).

Image acquisition and analysis for MPLSM

To track the transferred cells in the aorta, time series of z-stacks were obtained. Depending on the size and shape of the ATLO, its anatomical location and cell density, the imaged area was variable (from 180 µm x 180 µm to 362 µm x 362 µm), the z step was 1.2 or 2.0 µm, with a total depth of 16-36 stacks. Four-dimensional reconstructions were obtained using Volocity 5 software (Improvision, UK). The number of cells per imaged area was calculated. Objects were tracked for at least nine time points. To quantify cell behavior, we calculated the mean velocity for each cell as it moved along its track in addition to length covered and displacement (straight line distance from the first position in the track to the last). Correction for tissue drift was carried out using custom made software (SULSA, UK). Briefly, elastic fibres, visualized by autofluorescence, were used as reference static objects. The average displacement of the centres of mass of elastic fibres was calculated for each

time point and the movement was subtracted from the movement of all tracked cells(Beltman et al., 2009).

Serum lipid analyses

Serum cholesterol and triglyceride of lipoprotein fractions were analysed by ultracentrifugation as previously described (Engel et al., 2011).

Statistical analyses

To compare flow cytometry data or morphometry data of multiple mouse groups the generalized estimating equation model (GEE) was used. Since data sets consist of repeated measures per mouse, the GEE model takes the correlation of these measurements per individual mouse into account and provides robust estimates for the standard errors of the regression coefficients, i.e. that even under misspecification of the chosen correlation structure, inferences regarding the group differences are still unbiased, which is advantageous when compared to traditional linear regression models. The parameters of the GEE model were estimated with Software IBM SPSS Statistics 20.0. All measurements are expressed as means of *n* samples ± SEM and data were analyzed by two-tailed Student`s t-test, or ANOVA with Benjamini-Hochberg correction, or multiple testing (Bonferroni) or as stated in figure legends.

SUPPLEMENTAL REFERENCES

Beltman, J.B., Maree, A.F., and de Boer, R.J. (2009). Analysing immune cell migration. Nat Rev Immunol *9*, 789-798.

Engel, K.M., Schrock, K., Teupser, D., Holdt, L.M., Tonjes, A., Kern, M., Dietrich, K., Kovacs, P., Krugel, U., Scheidt, H.A., et al. (2011). Reduced food intake and body weight in mice deficient for the G protein-coupled receptor GPR82. PLoS One 6, e29400.

Futterer, A., Mink, K., Luz, A., Kosco-Vilbois, M.H., and Pfeffer, K. (1998). The lymphotoxin beta receptor controls organogenesis and affinity maturation in peripheral lymphoid tissues. Immunity *9*, 59-70.

Galkina, E., Kadl, A., Sanders, J., Varughese, D., Sarembock, I.J., and Ley, K. (2006). Lymphocyte recruitment into the aortic wall before and during development of atherosclerosis is partially L-selectin dependent. J Exp Med *203*, 1273-1282.

Gibson, V.B., Benson, R.A., Bryson, K.J., McInnes, I.B., Rush, C.M., Grassia, G., Maffia, P., Jenkinson, E.J., White, A.J., Anderson, G.*, et al.* (2012). A novel method to allow noninvasive, longitudinal imaging of the murine immune system in vivo. Blood *119*, 2545-2551.

Grabner, R., Lotzer, K., Dopping, S., Hildner, M., Radke, D., Beer, M., Spanbroek, R., Lippert, B., Reardon, C.A., Getz, G.S., *et al.* (2009). Lymphotoxin beta receptor signaling promotes tertiary lymphoid organogenesis in the aorta adventitia of aged *Apoe*$^{-/-}$ mice. J Exp Med *206*, 233-248.

Kobayashi, M., Inoue, K., Warabi, E., Minami, T., and Kodama, T. (2005). A simple method of isolating mouse aortic endothelial cells. J Atheroscler Thromb *12*, 138-142.

Millington, O.R., Brewer, J.M., Garside, P., and Maffia, P. (2010). Imaging interactions between the immune and cardiovascular systems in vivo by multiphoton microscopy. Methods Mol Biol *616*, 193-206.
