# Supplementary figures and images for: Artery Tertiary Lymphoid Organs Control Aorta Immunity and Protect against Atherosclerosis via Vascular Smooth Muscle Cell Lymphotoxin β Receptors
Source: Immunity. 2015 Jun 16;42(6):1100–15. doi: 10.1016/j.immuni.2015.05.015 (PMC4678289; doi:10.1016/j.immuni.2015.05.015)

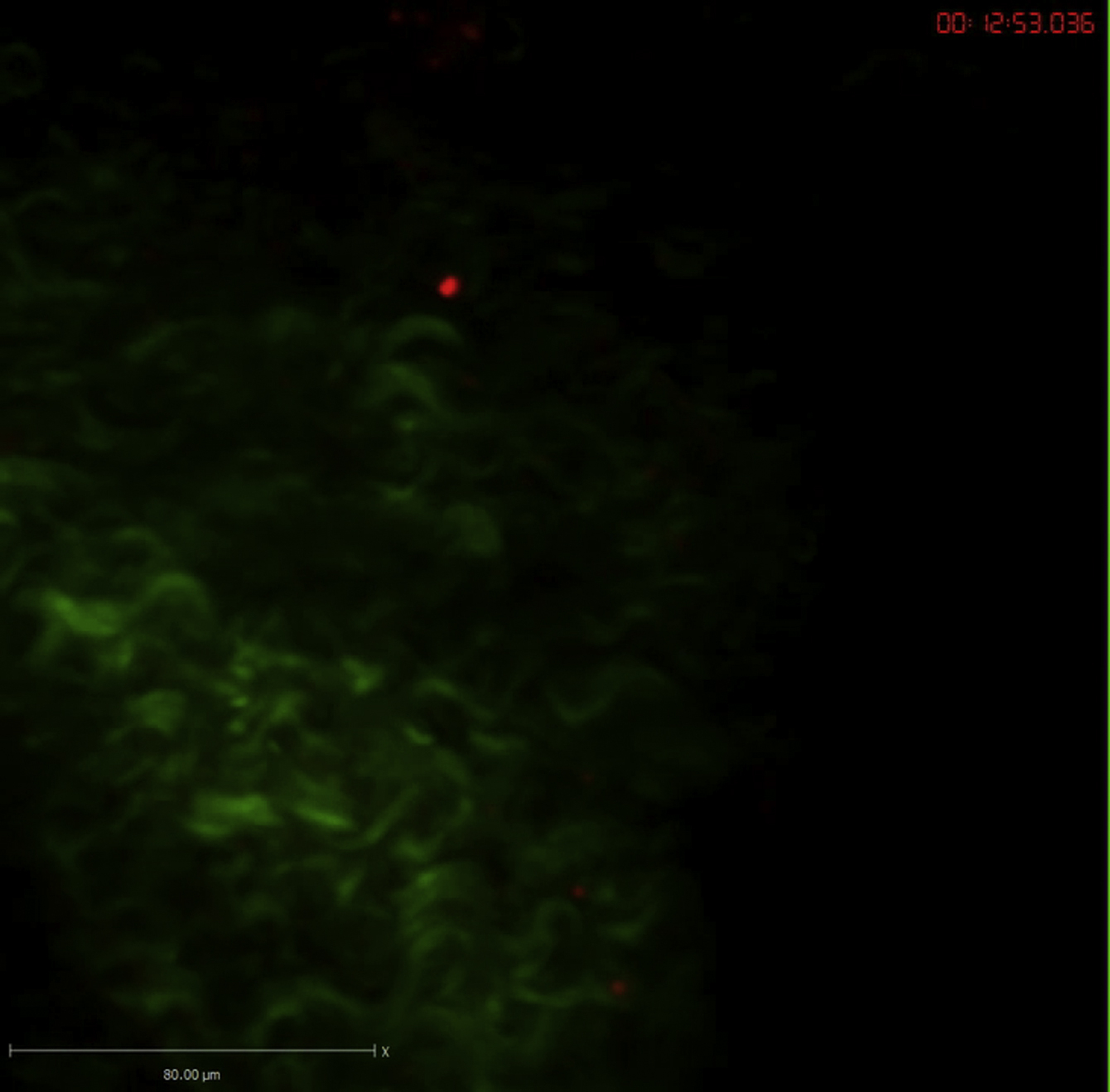

Supplement: Movie S1. Imaging of CMPTX-Labeled Cells in the Aged WT Aorta Adventitia, Related to Figure 3 [file mmc3.jpg]

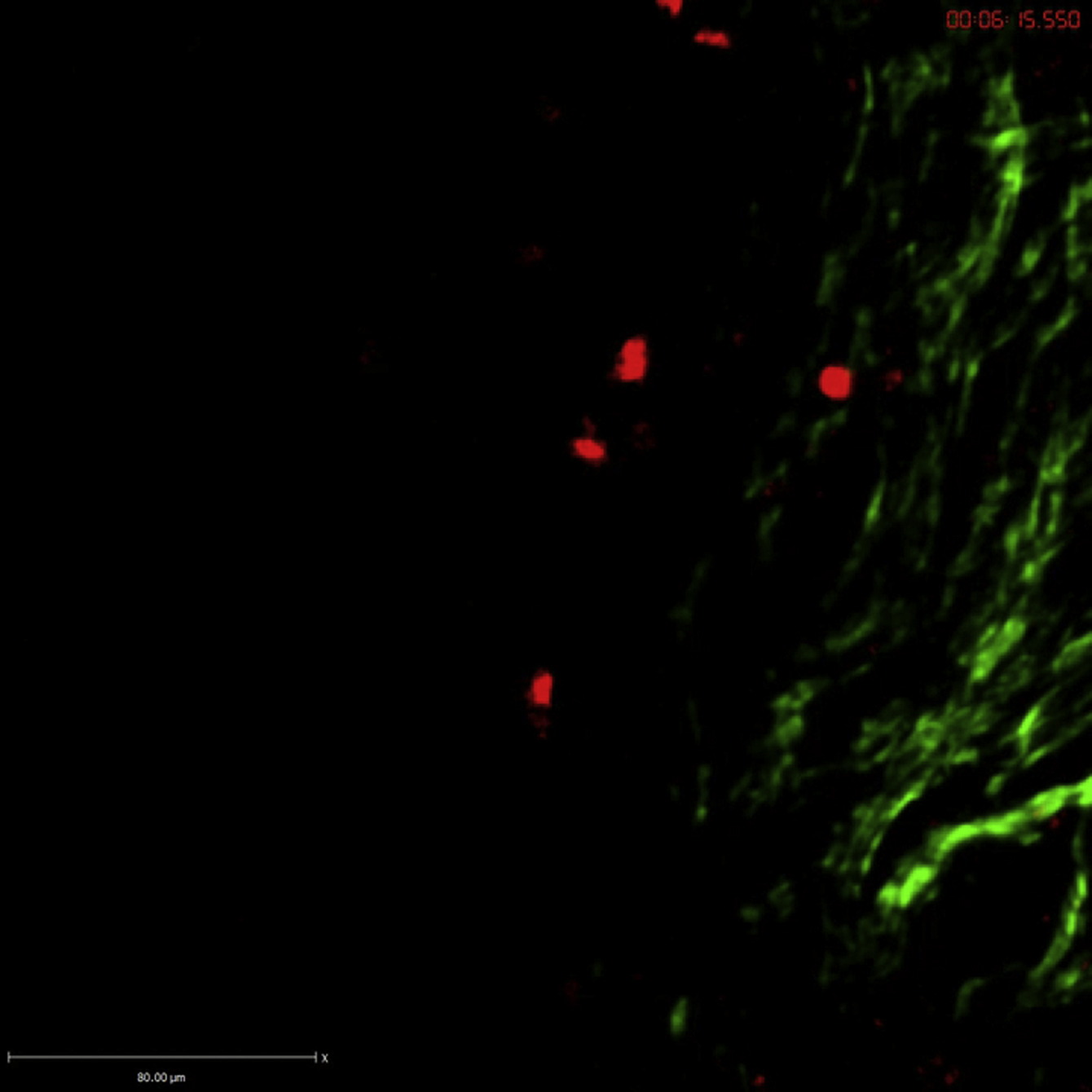

Supplement: Movie S2. Imaging of CMPTX-Labeled Cells in ATLO of aged Apoe−/− Abdominal Aorta Adventitia, Related to Figure 3 [file mmc4.jpg]

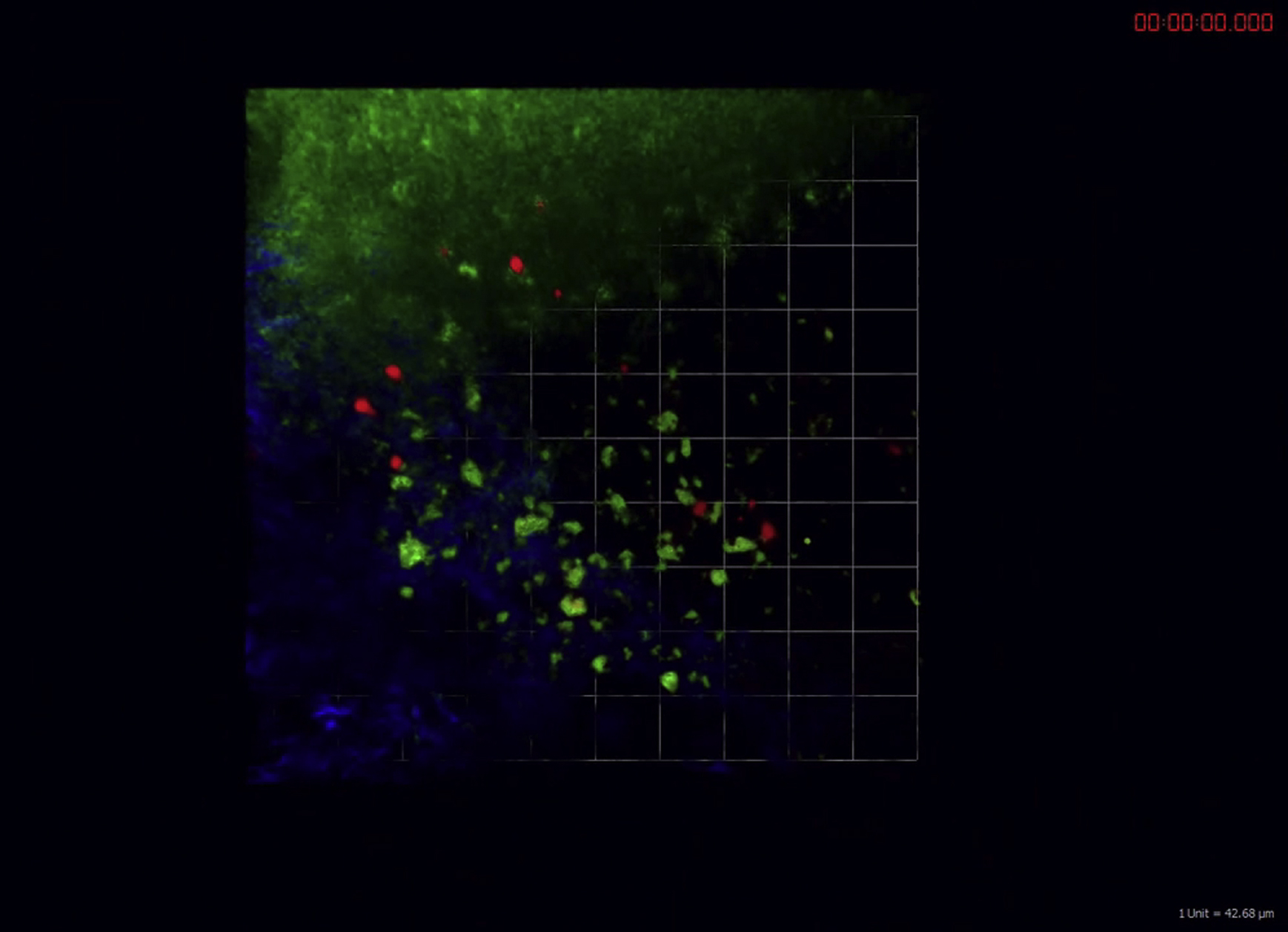

Supplement: Movie S3. Imaging of Antigen-Specific T-DC Clustering in ATLOs, Related to Figure 5 [file mmc5.jpg]

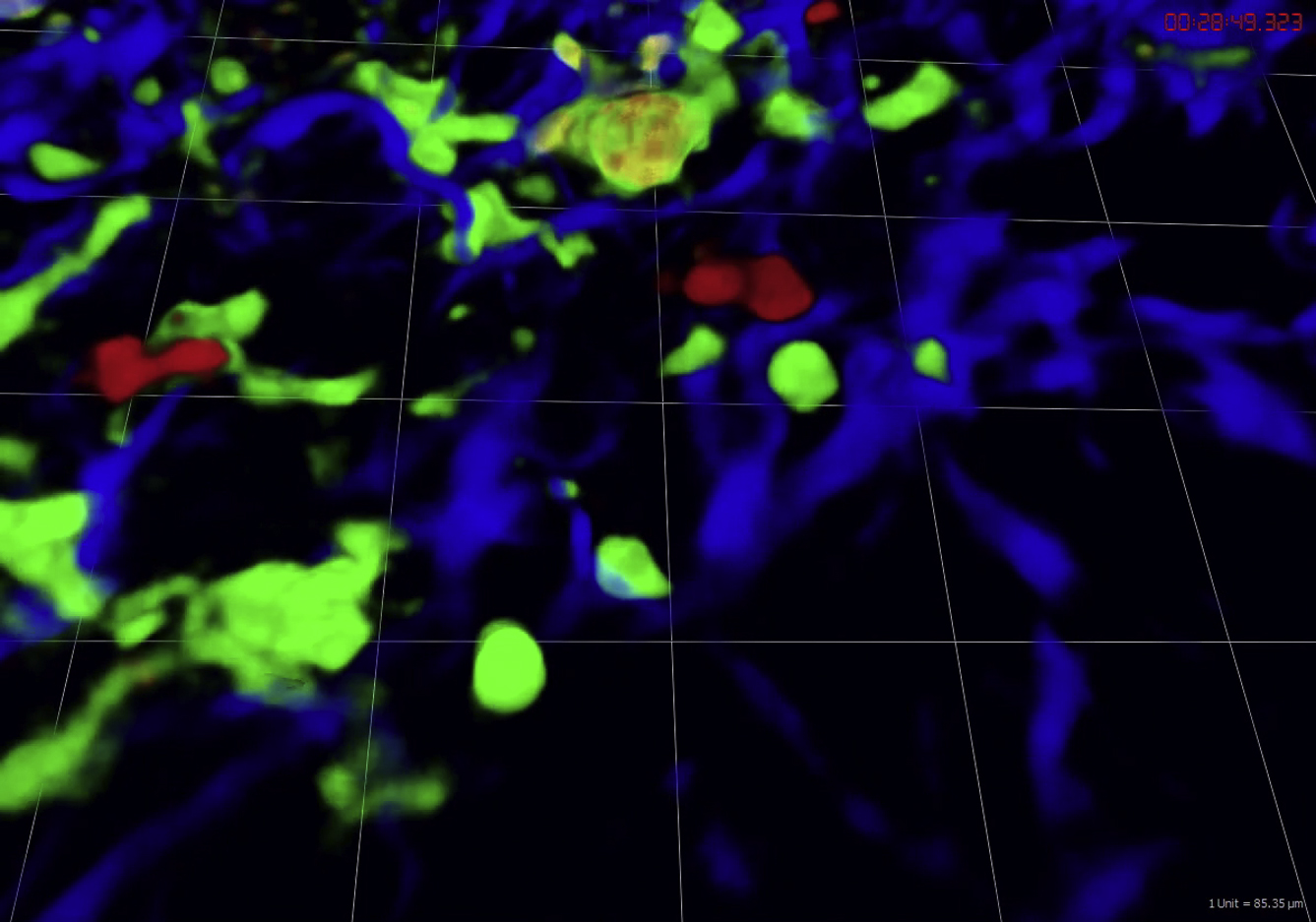

Supplement: Movie S4. High-Magnification Imaging of Antigen-Specific T-DC Clustering in ATLOs, Related to Figure 5 [file mmc6.jpg]
